# Simultaneous nitrosylation and N-nitrosation of a Ni-thiolate model complex of Ni-containing SOD†
†Electronic supplementary information (ESI) available: Synthetic and spectroscopic/reactivity details, and full crystallographic information for **2** and **3**. CCDC 1850485 and 1850488. For ESI and crystallographic data in CIF or other electronic format see DOI: 10.1039/c8sc03321h


**DOI:** 10.1039/c8sc03321h

**Published:** 2018-09-17

**Authors:** Phan T. Truong, Ellen P. Broering, Stephen P. Dzul, Indranil Chakraborty, Timothy L. Stemmler, Todd C. Harrop

**Affiliations:** a Department of Chemistry , Center for Metalloenzyme Studies , The University of Georgia , Athens , Georgia 30602 , USA . Email: tharrop@uga.edu; b Departments of Pharmaceutical Sciences, Biochemistry, and Molecular Biology , Wayne State University , Detroit , Michigan 48201 , USA; c Department of Chemistry and Biochemistry , Florida International University , Miami , Florida 33199 , USA

## Abstract

Nitric oxide (NO) is used as a substrate analogue/spectroscopic probe of metal sites that bind and activate oxygen and its derivatives. To assess the interaction of superoxide with the Ni center in Ni-containing superoxide dismutase (NiSOD), we studied the reaction of NO^+^ and NO with the model complex, Et_4_N[Ni(nmp)(SPh-*o*-NH_2_-*p*-CF_3_)] (**1**; nmp^2–^ = dianion of *N*-(2-mercaptoethyl)picolinamide; ^–^SPh-*o*-NH_2_-*p*-CF_3_ = 2-amino-4-(trifluoromethyl)benzenethiolate) and its oxidized analogue **1^ox^**, respectively. The ultimate products of these reactions are the disulfide of ^–^SPh-*o*-NH_2_-*p*-CF_3_ and the *S*,*S*-bridged tetrameric complex [Ni_4_(nmp)_4_], a result of S-based redox activity. However, introduction of NO to **1** affords the green dimeric {NiNO}^10^ complex (Et_4_N)_2_[{Ni(κ^2^-*S*Ph-*o-N*NO-*p*-CF_3_)(NO)}_2_] (**2**) *via* NO-induced loss of nmp^2–^ as the disulfide and N-nitrosation of the aromatic thiolate. Complex **2** was characterized by X-ray crystallography and several spectroscopies. These measurements are in-line with other tetrahedral complexes in the {NiNO}^10^ classification. In contrast to the established stability of this metal-nitrosyl class, the Ni–NO bond of **2** is labile and release of NO from this unit was quantified by trapping the NO with a Co^II^–porphyrin (70–80% yield). In the process, the Ni ends up coordinated by two *o*-nitrosaminobenzenethiolato ligands to result in the structurally characterized *trans*-(Et_4_N)_2_[Ni(*S*Ph-*o-N*NO-*p*-CF_3_)_2_] (**3**), likely by a disproportionation mechanism. The isolation and characterization of **2** and **3** suggest that: (i) the strongly donating thiolates dominate the electronic structure of Ni-nitrosyls that result in less covalent Ni–NO bonds, and (ii) superoxide undergoes disproportionation *via* an outer-sphere mechanism in NiSOD as complexes in the {NiNO}^9/8^ state have yet to be isolated.

## Abstract 2

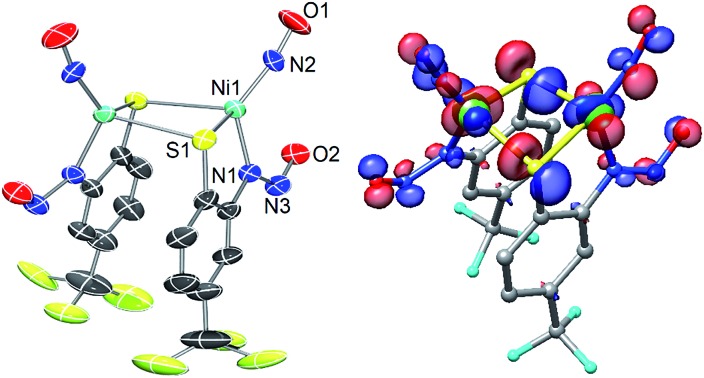
Nitric oxide reacts with a NiSOD model complex to yield a thiolate-ligated/N-nitrosated {NiNO}^10^ species with unusually labile Ni–NO bonds.

## Introduction

Nitric oxide (NO) and its derivatives (termed reactive nitrogen species or RNS) play a vital role in a variety of mammalian (and in some cases bacterial) physiological and pathological processes.^1^–^4^ Additionally, this gaseous free radical has applications in fundamental research, especially in bioinorganic chemistry, where it is utilized as a structural/spectroscopic probe of O_2_ (and other reactive oxygen species, *e.g.*, O_2_˙^–^ and H_2_O_2_) binding/activating metalloenzymes.^5^–^9^ In general, this approach is employed because metal-nitrosyl (MNO) bonds are highly covalent, and hence more stable, than metal–dioxygen (M–O_2_) adducts.^10^ The use of NO as an O_2_ analogue is based on similar electronic structures between these diatoms and their reduced derivatives.^3^ For example, ^3^NO^–^ (termed the nitroxyl anion), the one electron reduced analogue of NO, is isoelectronic with O_2_ with two unpaired π* electrons in the HOMO. Additionally, NO, while not isoelectronic with O_2_˙^–^, has the same ground state electronic structure with a singly occupied π* MO. Thus, NO interactions with the active sites of O_2_-activating/ROS-breakdown enzymes report coordination (inner-sphere substrate binding) and the extent of substrate bond activation from vibrational spectroscopic measurements of the N–O and M–NO stretching frequencies.

**Chart 1:**
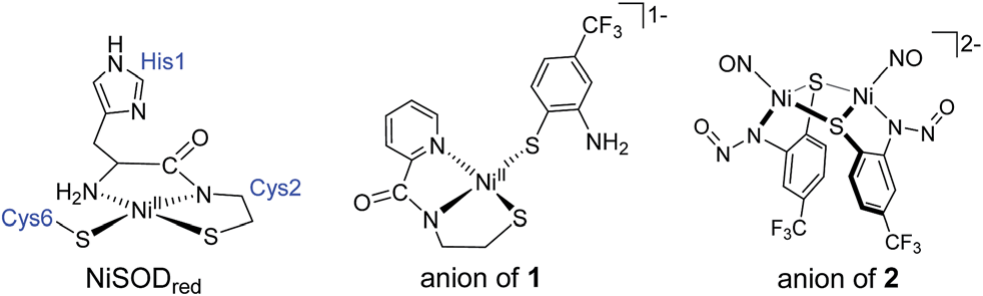
Structures of the active site of NiSOD_red_ (left; His1 coordinates to Ni^III^ in NiSOD_ox_), the anion of the NiSOD model complex Et_4_N[Ni(nmp)(SPh-*o*-NH_2_-*p*-CF_3_)] (**1**) (center; nmp^2–^ = dianion of the N_2_S ligand *N*-(2-mercaptoethyl)picolinamide), and the anion of the {NiNO}^10^ complex (Et_4_N)_2_[{Ni(κ^2^-*S*Ph-*o-N*NO-*p*-CF_3_)(NO)}_2_] (**2**).

Since 2009, our lab has designed and constructed numerous low molecular weight models of the active site of Ni-containing superoxide dismutase (NiSOD).^11^–^18^ NiSOD is an unprecedented SOD due to Ni^III/II^-coordination to cysteinato-S (CysS) and peptido-N donors (Chart 1), the former of which is susceptible to oxidative modification by the substrate (O_2_˙^–^) and products (O_2_ and H_2_O_2_) of the SOD catalyzed reaction.^19^,^20^ Few models employ ligands with the correct spatial disposition and electronic nature of the unique N_3_S_2_ donor set found in the active site.^21^–^23^ Moreover, fewer report reversible electrochemical and/or spectroscopic evidence for the Ni^III^ oxidation state due to redox associated with the coordinated thiolates. One model from our lab, namely Et_4_N[Ni(nmp)(SPh-*o*-NH_2_-*p*-CF_3_)] (**1**; nmp^2–^ = dianion of the N_2_S ligand *N*-(2-mercaptoethyl)picolinamide; see Chart 1) displays a reversible redox-event at –0.43 V (*vs.* Fc/Fc^+^ in DMF) that, based on EPR, UV-vis, MCD, and DFT computations, represents the electrochemical conversion from Ni^II^ in **1** to a Ni^II^-thiyl ↔ Ni^III^-thiolate resonance species termed **1^ox^**.^16^ Because substrate binding to Ni in NiSOD has not been defined, although most reports favor an outer-sphere mechanism,^15^,^24^ we were curious to use NO as an O_2_˙^–^ probe to define potential intermediates that may be traversed in the NiSOD mechanism. We report here, for the first time, the reactions and product characterization of NO (and NO^+^) with **1** and the well-defined analogue of NiSOD_ox_ (**1^ox^**). NO/NO^+^ oxidize the aromatic thiolate ligand in **1^ox^** and **1**, respectively. However, introduction of NO to **1** affords the green dimeric {NiNO}^10^ complex (Et_4_N)_2_[{Ni(κ^2^-*S*Ph-*o-N*NO-*p*-CF_3_)(NO)}_2_] (**2**) *via* NO-induced loss of nmp^2–^ as the disulfide and N-nitrosation of the aromatic thiolate (Chart 1). While **2** bears little resemblance to NiSOD, its formation indicates how reactive NiSOD models such as **1** are in the presence of redox-active diatoms and suggest similar paths for other biological Ni-thiolate sites. Additionally, **2** contains a labile Ni–NO bond, a new feature for the {NiNO}^10^ formulation that appears to be controlled by the presence of the thiolate ligands. We describe the synthesis, spectroscopy, electronic structure, reactivity and mechanistic insight into the formation of the Ni-nitrosyl in this account.

## Results and discussion

**Scheme 1:**
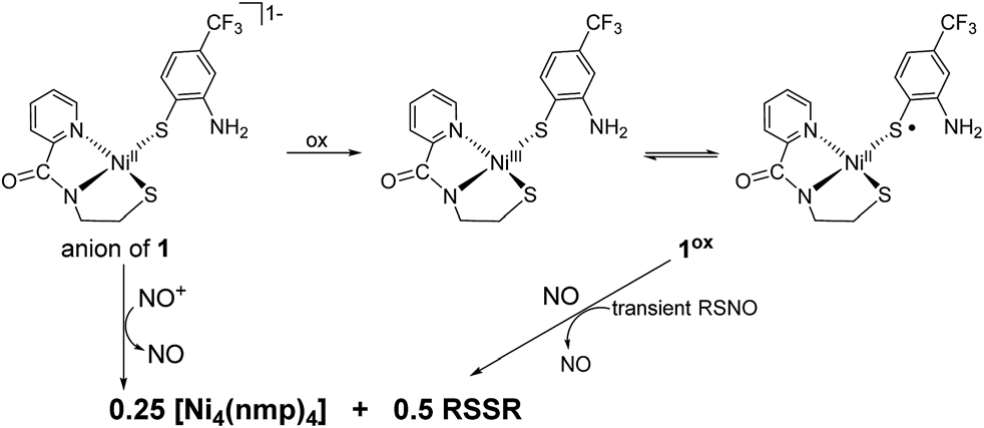
NO and NO^+^ reactions of **1** and **1^ox^**, respectively. R = 2-amino-4-(trifluoromethyl)benzenethiolate.

In the anticipation of isolating a Ni-nitrosyl as an analogue of a potential Ni-superoxo/peroxo catalytic intermediate of NiSOD, we examined the reaction of **1** with NOBF_4_ and *in situ* prepared **1^ox^** with NO (Scheme 1). In theory, both reactions yield the same product. For example, nitrosonium (NO^+^; a strong oxidant, *E* = +0.56 V *vs.* Fc/Fc^+^ in DMF^25^) will oxidize **1** to **1^ox^** and form NO in the process. The newly generated **1^ox^** (*S* = 1/2) then reacts with NO to form the Ni-nitrosyl, formally a {NiNO}^8^ complex, assuming binding of NO and no other coordination sphere changes, using the notation defined by Enemark and Feltham.^26^ Likewise, NO will readily intercept paramagnetic **1^ox^** to generate the same species. Mixing a DMF solution of **1** with NOBF_4_ (1 : 1) resulted in instantaneous bleaching of the solution, consistent with oxidation of the RS^–^ ligand to disulfide (RSSR), and the appearance of a dark-red precipitate that was spectroscopically identified to be the neutral *S*,*S*-bridged tetramer [Ni_4_(nmp)_4_] (Scheme 1).^16^ This outcome is typical for all [Ni(nmp)(SR)]^–^ complexes when treated with chemical oxidants, *i.e.*, S-oxidation of the coordinated monodentate thiolate to RSSR.^15^ Incidentally, the same result was obtained when introducing NO(g) into a DMF solution of *in situ* generated **1^ox^**. In this case, formation of the disulfide may traverse a fleeting, and yet to be characterized, RSNO intermediate that releases NO *via* homolytic cleavage of the RS–NO bond (Scheme 1).^27^ Overall, a Ni-nitrosyl was not isolated. This result may not be too surprising considering that all known Ni-nitrosyls are in the {NiNO}^10^ Enemark–Feltham (EF) classification,^28^ although a {NiNO}^9/8^ species is not entirely unrealistic in light of the strong donors present in **1** and in NiSOD, *i.e.*, peptido-N and alkyl-thiolato-S.

**Fig. 1:**
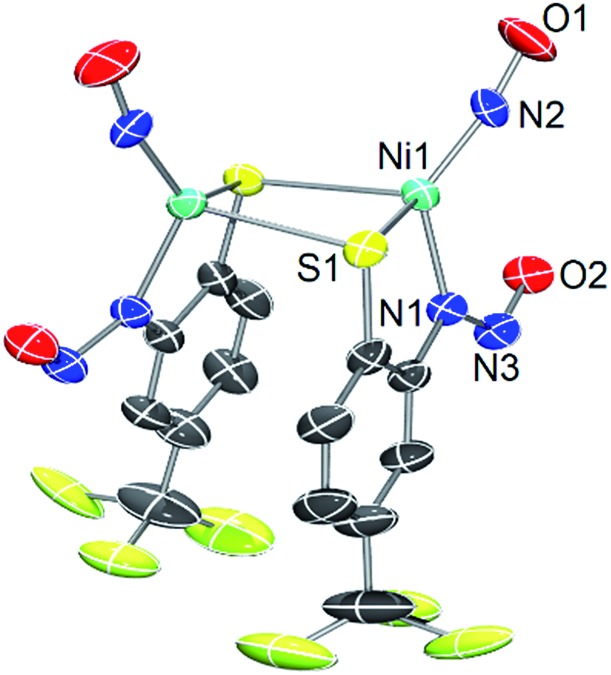
X-ray structure of the anionic portion of **2** with the atom labeling scheme (50% thermal probability). H atoms and Et_4_N^+^ counterions are omitted for clarity.

As a control, we also explored the reaction of Ni^II^ complex **1** with NO. In general, NO does not react with square-planar [Ni(nmp)(SR)]^–^ (R = simple aryl or alkyl groups) complexes due to their diamagnetic nature. However, when R contains a potentially bidentate chelate, as in **1**, a different course takes place. For instance, exposing a DMF solution of **1** with NO(g) for 30 s resulted in a gradual change of the solution from dark-red to green over several minutes. Workup of this reaction indicated a Ni-nitrosyl based on the strong double-humped peak in the N–O stretching (*ν*_NO_) region of the IR spectrum (*vide infra*). Subsequent crystallization of the bulk material from MeCN/Et_2_O at –20 °C resulted in green crystals of a dinuclear thiolate-bridged {NiNO}^10^ complex (Et_4_N)_2_[{Ni(κ^2^-*S*Ph-*o-N*NO-*p*-CF_3_)(NO)}_2_] (**2**) as depicted in Fig. 1 with selected metric parameters listed in Table 1. The Ni centers in **2** are distorted tetrahedral (*τ*_4_ = 0.73 (ref. 29)) resulting from N_2_S_2_ coordination of the thiolato-S/deprotonated amine-N of the S-bridged *o*-nitrosaminobenzenethiolate and a terminal nitrosyl. To our knowledge, complex **2** represents the first example of a structurally characterized first-row metal complex with both a coordinated nitrosyl and amine-N-bound nitrosamine. In accord with other tetrahedral {NiNO}^10^ complexes,^28^ the Ni–N(O) distance is short (1.659 Å), the N–O bond (1.182 Å) is intermediate between free NO˙ (1.15 Å) and ^1^HNO (1.21 Å),^10^ and the Ni–N–O bond angle is close to linear albeit slightly bent (167.8°) (see Table 1). Complex **2** is analogous to the limited number of four-coordinate/S-bound Ni-nitrosyls,^30^–^34^ fewer of which contain Ni–S_thiolate_ bonds^31^,^33^ which display Ni–N(O) (1.663–1.683 Å), N–O (1.131–1.173 Å), and Ni–N–O (156.6–173.9°) distances/angles in similar ranges. Even neutral/cationic P-35–40 and N-bound^34^,^41^–^43^ L_3_Ni–NO/L_2_XNi–NO complexes exhibit similar metric parameters. The coordinated nitrosamine is bent (N–N–O: 115.4°), *i.e.*, sp^2^-hybridized nitroso-N, with N–N and N–O distances of 1.299 and 1.269 Å, respectively. These values suggest a small degree of delocalization in the R–N–N–O unit. However, the structure is more biased towards the nitrosamino R–N^–^–N=O *versus* diazoate R–N=N–O^–^ resonance form. To compare, the structure of *syn*-methanediazoate (N–N: 1.246 Å, N–O: 1.306 Å) reflects the true double bond character in an authentic R–N=N–O unit.^44^ These values are somewhat comparable to other N-bound nitrosamine complexes,^45^,^46^ especially [CpNi(PPh_3_)(ON_2_Ph-*p*-NO_2_)] (**I**)^47^ (N–N: 1.327 Å, N–O: 1.249 Å, N–N–O: 113.1°). Structures of coordinated nitroso-N-metal complexes (*vs.* amine-N as in **2**) also afford similar structural parameters in the RNNO.^48^ In contrast, O-bound nitrosamine complexes appear to favor more of a resonance delocalized structure as the N–N (1.275–1.288 Å) and N–O (1.251–1.275 Å) distances in a series of [Fe^III^(P)(ONNR_2_)_2_]^+^ (P = porphyrin) complexes are nearly identical and result in a single ^15^N-sensitive peak in the IR due to overlapping *ν*_NN_/*ν*_NO_ modes.^49^–^51^

**Table 1:** Selected bond distances (Å) and bond angles (deg) from the X-ray crystal structure of **2**, compared with the DFT-optimized model 2*

	X-ray structure **2**	DFT (BP86/def2-TZVPP) optimized structure 2*
Ni1–S1	2.3169(7)	2.294
Ni1–S1′	2.3555(6)	2.344
Ni1–N2	1.659(7)	1.648
Ni1–N1	1.971(2)	1.974
N2–O1	1.182(8)	1.191
N1–N3	1.299(3)	1.324
N3–O2	1.269(3)	1.254
S1–Ni–S1′	96.66(2)	90.05
S1–Ni–N1	86.21(6)	86.67
S1′–Ni–N1	99.10(6)	98.40
S1–Ni–N1	123.8(5)	125.92
N2–Ni–S1′	109.8(6)	113.43
N1–Ni–N2	133.6(5)	132.15
Ni1–N2–O1	167.8(12)	171.42
N1–N3–O3	114.2(2)	115.80
*τ*_4_	0.73	0.72

**Fig. 2:**
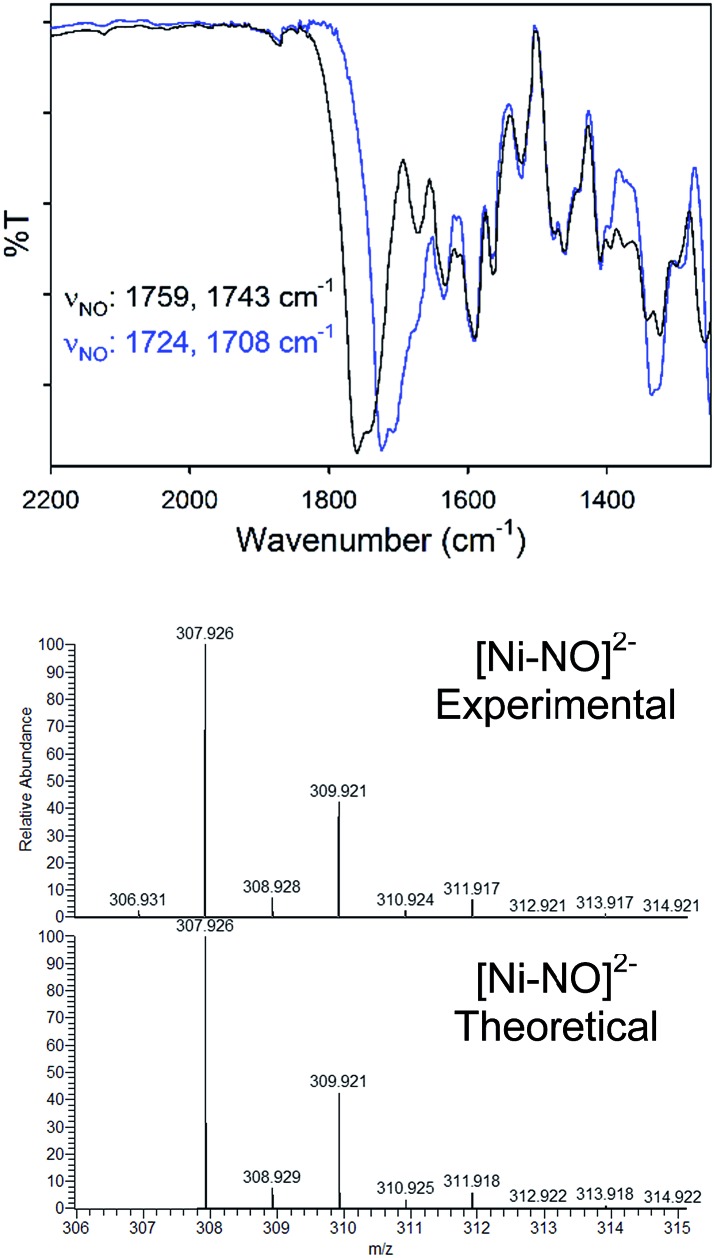
(Top) Solid-state IR of the *ν*_NO_ region for **2** (black) and **2-^15^NO** (blue) in a KBr matrix. (Bottom) High resolution ESI-MS(–) of **2** with the theoretical isotopic distribution.

Complex **2** was characterized by a variety of spectroscopic methods. The solid-state IR spectrum (KBr matrix) of **2** exhibits two closely spaced, but well-resolved, *ν*_NO_ at 1759 and 1743 cm^–1^ (1724, 1708 cm^–1^ for **2-^15^NO**; Δ*ν*_NO_: 35 cm^–1^; see Fig. 2). These values fall in the range of known tetrahedral, neutral, and anionic {NiNO}^10^ complexes.^28^ Because **2** is of *C*_2_ symmetry (*cis* NO, *syn* bridging thiolates), two IR-active N–O vibrational modes are expected. The other feasible isomer of **2** would be of *C*_*i*_ symmetry (*trans* NO, *anti* bridging thiolates) and would display one IR-active N–O stretch. Indeed, the IR spectrum of **2** in DMSO exhibits one *ν*_NO_ at 1784 cm^–1^ suggesting possible *cis*/*trans*-NO conversion in solution (or an averaged *ν*_NO_ value due to rapid tumbling) or thiolate-bridge splitting to yield a four-coordinate mononuclear {NiNO}^10^ with DMSO as the fourth ligand, *i.e.*, [Ni(κ^2^-*S*Ph-*o-N*NO-*p*-CF_3_)(DMSO)(NO)]^–^. The ^1^H NMR spectrum of **2** in CD_3_CN (Fig. S6†) or DMSO-d_6_ (not shown) are similar and thus do not distinguish any of the proposed structures. Comparable IR spectral changes in the opposite direction are observed for the one other thiolate-supported anionic dinuclear {NiNO}^10^, (Et_4_N)_2_[Ni_2_(NO)_2_(μ-SPh)_2_(SPh)_2_] (**II**), with *trans* NO ligands (*ν*_NO_: 1709 cm^–1^ in KBr; 1751, 1721 cm^–1^ in THF).^33^ A similar situation is described for a pyrazolate-bridged anionic dinuclear {NiNO}^10^ complex.^52^ IR peaks arising from the nitrosamine were not as obvious due to multiple overlapping peaks in the region (Fig. S5†). However, ^15^N-sensitive peaks in the IR of **2** at 1342 and 1258 cm^–1^ (1326, 1249 cm^–1^ in **2-^15^NO**) are assigned as *ν*_NO_ and *ν*_NN_, respectively. In comparison, a series of secondary nitrosamines display *ν*_NO_: 1428–1463 cm^–1^ and *ν*_NN_: 1035–1154 cm^–1^ in CCl_4_.^53^ Therefore, a significant degree of delocalization occurs in the RNNO unit of **2** to cause the corresponding downshift in *ν*_NO_/upshift in *ν*_NN_. While no paramagnetically shifted resonances are observed in the ^1^H NMR (CD_3_CN) of **2**, several species are indicated in freshly prepared solutions (Fig. S6†) that are likely caused by the lability of the Ni–NO bond and presence of nmpS_2_ (*vide infra*). The ^15^N NMR spectrum of **2-^15^NO** confirms multiple solution speciation with four major peaks in the range for nitrosamines and linearly coordinated NO (*δ*: 40–190 ppm in CD_3_CN, *vs.* CH_3_NO_2_, Fig. S7†).^54^–^57^ Moreover, the ^1^H NMR of thiolate-bridged dinuclear complex **II** displays broadened aryl-H resonances caused by rapid exchange of PhS^–^ ligands because of disproportionation to the mononuclear (Et_4_N)_2_[Ni(NO)(SPh)_3_] (**III**) and an uncharacterized [Ni(NO)(SPh)] species.^33^ However, high-resolution electrospray ionization mass spectrometry (HR-ESI-MS; negative mode) displays one dominant compound with the formula and isotopic distribution consistent with the dianionic portion of **2** (*m*/*z*: 307.926, *z* = 2, Fig. 2, S8 and S9†) and **2-^15^NO** (*m*/*z*: 309.920, *z* = 2; Fig. S10 and S11†), although this measurement does not discriminate against *cis* and *trans* NO conformers. Another minor peak in the HR-ESI-MS(–) is centered at *m*/*z*: 248.960 (*z* = 2; Fig. S8†) that suggests a new [Ni(N_2_S_2_)]^2–^ species through loss of the Ni-coordinated NO and one Ni (*vide infra*).

**Fig. 3:**
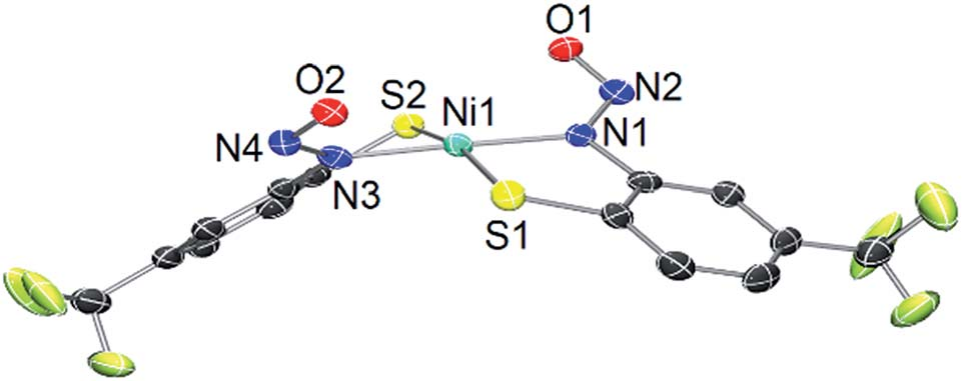
X-ray structure of the dianion of **3** with the atom-labeling scheme (50% thermal probability). One of two crystallographically distinct molecules shown. H atoms, Et_4_N^+^ counterions, and solvent of crystallization (Et_2_O) are omitted for clarity.

Solutions of **2**, especially in donor solvents such as MeCN or DMF, gradually lose their green color to give red-brown solutions more reminiscent of square-planar Ni^II^–N_2_S_2_ complexes.^15^,^58^ Even freshly prepared CD_3_CN solutions of **2** exhibit multiple peaks in the ^1^H/^15^N NMR, and ESI-MS shows a new species with a Ni isotope pattern at *m*/*z* ∼ 249 (*vide supra*). This change is enhanced when vacuum is applied and FTIR spectra of these reaction mixtures lack any *ν*_NO_ suggesting the loss of coordinated NO from **2** to generate a new Ni species. Slow diffusion of Et_2_O into MeCN solutions of **2** that have been left standing for several weeks result in crystals (10–20% isolated yield from crystallization) of a square-planar (*τ*_4_ = 0.12) Ni^II^ compound where two N,S-chelating *o*-nitrosaminobenzenethiolato ligands bind to Ni in a *trans* configuration, *viz. trans*-(Et_4_N)_2_[Ni(*S*Ph-*o-N*NO-*p*-CF_3_)_2_] (**3**) (Fig. 3). The bond lengths (Ni–S: 2.2072 Å, Ni–N: 1.896 Å) and angles (Table S3†) are similar to other planar Ni^II^–N_2_S_2_ complexes that contain κ^2^-N,S-*o*-aminobenzenethiolate ligands.^59^–^61^ The Ni–N distance in **3** is shorter than the typical Ni–N_amine_ bond and reflects the enhanced donor strength of the deprotonated nitrosamino-N, which is comparable to, although weaker than, a Ni–N_carboxamido_ (∼1.86 Å).^15^,^58^ No evidence for a coordinated ligand radical is evident from the X-ray structure (*i.e.*, short C–S, C–N distances of the coordinated *o*-aminobenzenethiolate^62^) and confirm the N,S-ligand is a closed-shell dianion. The R–N–N–O linkage in **3** (avg. of two crystallographically distinct molecules, N–N: 1.309 Å, N–O: 1.264 Å; avg. N–N–O: 115.2°) is unremarkable from **2**. ^1^H and ^15^N NMR (RN^15^*N*O, *δ*: 194 ppm *vs.* CH_3_NO_2_) of crystals of **3** are consistent with the X-ray structure and analogous to other nitrosamines (Fig. S13 and S14†).^55^,^57^ As expected, the IR of **3** lacks the intense *ν*_NO_ from the NiNO of **2** (although IR and ESI-MS show that some **2** remains even in crystals of **3**, Fig. S12†) and the *ν*_NO_ and *ν*_NN_ of the R–N–N–O unit is similar. HR-ESI-MS(–) confirm this formulation with peaks corresponding to [M–2Et_4_N]^2–^ (*m*/*z*: 248.960, *z* = 2, for **3**; *m*/*z*: 249.957, *z* = 2, for **3-^15^NO**) as the prominent peak (Fig. S15–S18†).

To confirm that NO(g) is released from **2** (forming **3** among other products), solutions of **2** were mixed with the NO(g) trap [Co(T(-OMe)PP)] (T(-OMe)PP = 5,10,15,20-tetrakis(4-methoxyphenyl)-21*H*,23*H*-porphine).^63^ For example, mixing **2** and the Co^II^–P (1 : 2) in CH_2_Cl_2_ at RT for 24 h resulted in the {CoNO}^8^ complex [Co(T(-OMe)PP)(NO)] in ∼70% avg. yield as quantified by ^1^H NMR (CD_2_Cl_2_) and further verified by IR spectroscopy using **2-^15^NO** (Fig. S21–S23†). Notably, the reaction mixture becomes red over the course of the reaction. Workup of this solution after separating the Co–P compounds (MeOH-insoluble) reveals the presence of **3** (MeOH-soluble) *via*^1^H NMR to confirm the fate of the {NiNO}^10^ complex **2**. To eliminate bimolecular NO-transfer *via* a putative Co···NO···Ni intermediate, NO(g) release was further verified by vial-to-vial trapping reactions wherein a CH_2_Cl_2_ solution of the Co^II^–P was separated from an MeCN solution of **2** (Co^II^–P in excess, see the ESI†). Carrying out this reaction confirmed that NO(g) is indeed released from **2** (or **2-^15^NO**) to generate the {CoNO}^8^ porphyrin complex (80% avg. yield) as shown by ^1^H NMR and IR measurements (Fig. S24†). In contrast, no reaction takes place between THF solutions of **2** with [Fe(TPP)Cl] (1 : 2; TPP = 5,10,15,20-tetraphenylporphyrin), a common HNO (or NO^–^) trap.^64^ Although {NiNO}^10^ has not been characterized as a particularly labile EF notation, we note that the majority of these complexes are cationic or neutral without coordinated thiolate ligands.^28^ Indeed, the thiolate-ligated {NiNO}^10^ complex **III** photochemically releases NO to [Co(TPP)] in MeCN suggesting some lability in the Ni–NO bond. Furthermore, the RN–NO bond is quite stable (as noted by formation of **3**) and the energetically stabilized MOs that contribute to the electronic structure of **2** and **3** where HOMO–3 represents a bonding MO with primary contributions from σ-NR and σ-NO orbitals (Fig. S25†).

**Fig. 4:**
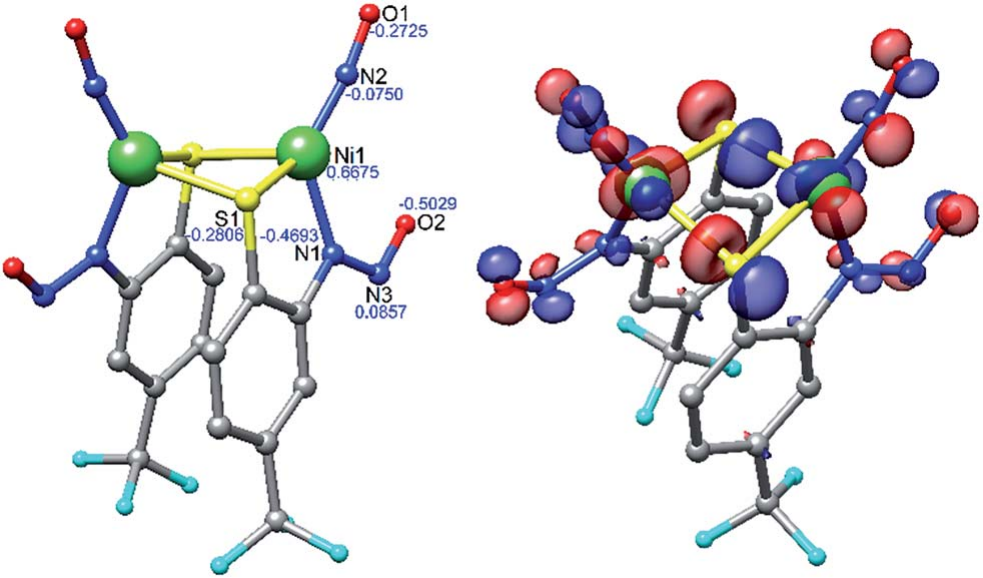
DFT (OLYP/def2-TZVPP) optimized structure of 2* (left) with natural population analysis charges in blue and HOMO (right).

Density functional theory (DFT) computations have provided a deeper understanding of the electronic structure of a variety of metal nitrosyls,^65^,^66^ and we have employed them here for **2** and **3** at the OLYP/def2-TZVPP level of theory. Pure functionals such as BP86 and OLYP were used for geometry optimization and single point energy calculations, respectively, as these functionals have been established to deliver better matches with experimental geometries in MNO systems.^67^–^69^ Geometry optimization of **2** was performed with coordinates from the crystal structure to yield DFT-optimized complex 2* (Fig. 4, Tables 1, S5 and S7 in the ESI†). Structurally, 2* replicates the metrics of **2** well, suggesting the computational model is reasonable. While the distances in 2* are within ±0.025 Å of experimental values, the bond angles (especially S–Ni–S: –6.6°, and Ni–N–O: +3.6° from **2**) are slightly beyond the allowable tolerances for satisfactory DFT performance in small molecules (*i.e.*, distances ±0.03 Å; angles ±1°).^70^ However, these rules may be broken to some degree because of the enhanced complexity arising from the covalent MNO unit in **2**. The computations also reasonably match the two closely spaced N–O stretching frequencies for the symmetric and asymmetric *ν*_NO_ in the IR at 1730 and 1708 cm^–1^, respectively. The ∼30 cm^–1^ downshift from **2** is likely due to a slight overestimation of Ni–NO bond covalency arising from Ni-dπ backbonding. Previous calculations on three-71 and four-coordinate^43^,^72^ {NiNO}^10^ complexes support a Ni^II^–^3^NO^–^ (*S*_tot_ = 0, antiferromagnetically coupled) oxidation state assignment. This is comparable to high-spin nonheme {FeNO}^7^ systems that are classified as Fe^III^–^3^NO^–^ (*S*_tot_ = 3/2).^6^,^66^,^73^ In the Fe case, ^3^NO^–^ serves as a strong π-donor to afford a highly covalent Fe–NO bond.^74^ The strength of this interaction originates from the effective nuclear charge on the metal, which is controlled by the basicity of the supporting ligands.^75^ Thus, electron rich supporting ligands attenuate the π-basicity of ^3^NO^–^ to result in diminished M–NO bond covalency. This property has been established in the {FeNO}^7^ case, but not yet for {NiNO}^10^. Indeed, examination of the frontier MOs of 2* show that, much like other {NiNO}^10^ systems with Tp ligands^43^,^72^ (Tp = tris(pyrazolyl)borate), the LUMO is a π* MO primarily comprised of antibonding interactions between Ni-dπ and NO-π* orbitals (Fig. S25†). On the other hand, the HOMO (Fig. 4) and HOMO–1 have little contribution from NO, but large contributions from Ni-dσ (38.0%) and S-pσ (19.3%) orbitals of the Ni(μ-SR)_2_Ni core. The HOMO is antibonding in nature and suggests a thermally unstable structure. As expected from analogous {FeNO}^7^ systems, based on the increased donor strength of the anionic nitrosamine-N/thiolate-S supporting ligands in 2*, the covalency in the Ni–N–O unit is less than in TpNi–NO complexes and rationalizes the observed lability of the Ni–NO bond and the Ni(μ-SR)_2_Ni core in **2**.

DFT computations on 3* were performed in the same fashion as for 2*. Geometry optimized 3* is square-planar (*τ*_4_ = 0.09) with metric parameters on-par with the X-ray structure of **3** and within the error of the DFT method (Table S9†). Unlike 2*, the π* HOMO of 3* is comprised primarily of Ni(dπ)/S(pπ) contributions (Fig. S26†), typical of planar Ni^II^–N_2_S_2_ complexes with strong-field ligands and suggests a highly covalent Ni–SR bond.^15^

**Scheme 2:**
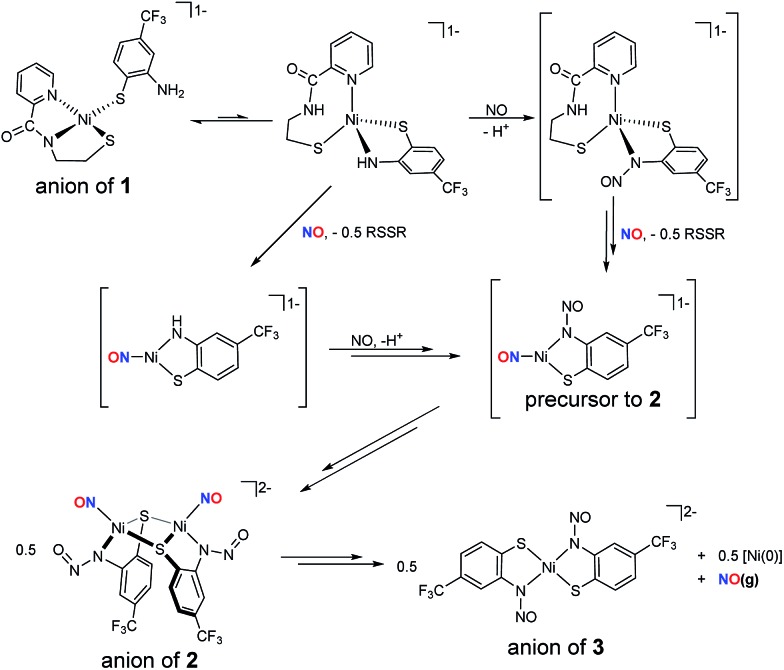
Working model for the formation of {NiNO}^10^ complex **2** and Ni^II^–N_2_S_2_ complex **3** starting from Ni^II^–N_2_S_2_ monomer **1**. RSSR: disulfide of nmp^2–^, *i.e.*, *N*-(2-mercaptoethyl)picolinamide. Intermediates represented in brackets have not been spectroscopically identified. The py-N of RSSR is a possible H^+^ receptor.

The formation of **2** likely follows a mechanistic path analogous to those observed in the reductive nitrosylation of Cu–amine systems, where one-equiv. of NO reacts with Cu^II^–NR_2_ complexes to yield R_2_N–NO and deligated Cu^I^.^76^,^77^ The difference here is that the nitrosated ligand remains coordinated and the resulting paramagnetic Ni binds NO radical. Our working model is depicted in Scheme 2. Complex **1** is likely in resonance with a distorted tetrahedral species which places the anilido-N in the coordination sphere. This proposal is supported by the presence of low intensity peaks in the ^1^H NMR spectrum of **1** and may explain the difficulty in crystallizing this complex.^16^ On the other hand, X-ray absorption spectroscopic (XAS) characterization of **1**, not reported previously, suggests a four-coordinate planar Ni^II^ center (XANES analysis, see Fig. S3†) with two O/N- and S-ligands at 1.90 Å and 2.17 Å (EXAFS, Fig. S3, Table S4†), respectively. Thus, **1** is structurally analogous to other [Ni(nmp)(SR)]^–^ complexes at least in the solid-state. Introduction of NO(g) can then result in either: (i) reduction of Ni^II^ to Ni^I^ and formation of NO^+^ that nitrosates the coordinated amine, or (ii) nitrosylation of Ni to yield {NiNO}^10^ with the electron originating from the coordinated thiolate of nmp^2–^ to result in the disulfide. Our results do not differentiate either of these transformations, but the disulfide of nmp^2–^ (*i.e.*, nmpS_2_^1^H NMR and IR of the reaction mixture, see Fig. S19 and S20†) is spectroscopically observed in the reaction mixture and checked against independently synthesized nmpS_2_. Thus, the fate of one proton and one electron is reasonably confirmed. At this point these intermediates can react with another equiv. of NO to yield the three-coordinate precursor to **2**. Compound **3** forms through either disproportionation (shown in Scheme 2) to yield a Ni^0^ species or ligand rearrangement *via* the loss of a Ni^I^–NO fragment (not shown). In ligand rearrangement, the products would be a Ni^I^–N_2_S_2_ precursor to **3** (**3-PC**), an L–Ni^I^–NO species (L = solvent), and free NO. Ultimately this Ni^I^ intermediate oxidizes **3-PC** to generate Ni^II^ complex **3** and an L–Ni^0^–NO complex that would presumably release NO(g) as evidenced by the NO(g) trap experiments (*vide supra*). While the reaction mechanism for the conversion of **2**-to-**3** is likely more complex, similar chemistry has been proposed for N-heterocyclic carbene (NHC) Ni-nitrosyls.^28^,^78^ The details of this mechanism are still under investigation.

## Conclusions

In conclusion, NiSOD model complex **1** reacts with NO(g) in the Ni^II^ state to form the metastable {NiNO}^10^ dimeric complex **2***via* loss of the nmp^2–^ ligand as the disulfide and N-nitrosation of the *o*-aminobenzenethiolate ligand. Reaction of NO with **1^ox^**, or NO^+^ with **1**, only yields the *S*,*S*-bridged tetrameric compound [Ni_4_(nmp)_4_] through oxidation of the aromatic thiolate ligand. While any reaction with NO (*S* = 1/2) is generally unexpected for square-planar (*S* = 0) Ni^II^ complexes, this Ni-nitrosyl likely forms due to an equilibrium mixture of **1** and a tetrahedral (*S* = 1) or five-coordinate derivative (Scheme 2). Even if NO were to result in an nmp-bound Ni–NO complex, the resulting {NiNO}^9^ (reaction of **1** with NO) or {NiNO}^8^ (reaction of **1^ox^** with NO) oxidation levels have yet to be defined and support an outer-sphere superoxide interaction in NiSOD. Although these EF notations have yet to be accessed, one would propose that NiSOD mimetics, especially with strong-field carboxamido-N and alkyl-thiolato-S donors, would surely stabilize such an electron poor species. Furthermore, the properties of complexes such as **2** extend to biology, where analogous S-bridged mononitrosyl species, *i.e.*, Fe–S clusters and tetrahedral (RS)_3_Fe–NO complexes are proposed as intermediates in the repair of NO-damaged clusters.^79^–^81^ Complex **2** is stable in the solid-state but breaks down slowly in solution causing rupture of the Ni(μ-SR)_2_Ni core and release of NO that was trapped in near quantitative yield with a Co^II^–porphyrin receptor. The resulting Ni^II^–N_2_S_2_ complex **3** (coordination of two *o*-aminobenzenethiolate in *trans* configuration) was isolated and structurally/spectroscopically characterized as the ultimate Ni breakdown product with the nitrosamine unit still intact. This release may take place through a disproportionation mechanism (or through ligand rearrangement), as has been proposed in other Ni-nitrosyls, to a yet ill-defined Ni^0^ complex (see Scheme 2).^28^,^78^ Hence, thiolate-supported {NiNO}^10^ cores are reactive. While nitrosamines have been utilized as sources of NO, the RN–NO homolytic bond dissociation energy (BDE) is high (87.7 kcal mol^–1^ (ref. 82)) compared to more traditional small molecule sources of NO such as nitrosothiols (RSNO) that have RS–NO BDEs between 20–32 kcal mol^–1^.^83^,^84^ Overall, the electronic structures of {NiNO}^10^ complexes are modulated by the supporting ligands. Indeed, the majority are stable entities; however, a small number are reactive and result in release of NO (thiolate-supported/anionic complexes **2** and **III**) or generate other reactive intermediates of environmental significance such as hyponitrite (N_2_O_2_^2–^) in five-coordinate {NiNO}^10^ species^85^ (highly reduced NO, with a severely bent Ni–N–O angle = 130°).

## Conflicts of interest

There are no conflicts to declare.

## Supplementary Material

Supplementary information

Crystal structure data
