# Supplementary material for: Simultaneous nitrosylation and N-nitrosation of a Ni-thiolate model complex of Ni-containing SOD
Source: Chem Sci. 2018 Sep 17;9(45):8567–74. doi: 10.1039/c8sc03321h (PMC6253683; doi:10.1039/c8sc03321h)
Supplement: Supplementary file 1 [file SC-009-C8SC03321H-s001.pdf]

Electronic Supplementary Material (ESI) for Chemical Science.
This journal is © The Royal Society of Chemistry 2018

Supporting Information for:

Simultaneous nitrosylation and N-nitrosation of a Ni-thiolate model complex of Ni-containing SOD

Phan T. Truong,[a] Ellen P. Broering,[a] Stephen P. Dzul,[b] Indranil Chakraborty,[c] Timothy L. Stemmler,[b] and Todd C. Harrop*,[a]

[a]Department of Chemistry and Center for Metalloenzyme Studies, The University of Georgia, 140 Cedar Street, Athens, Georgia 30602, United States
[b]Department of Pharmaceutical Sciences, Biochemistry, and Molecular Biology, Wayne State University, Detroit, Michigan 48201, United States
[c]Department of Chemistry and Biochemistry, Florida International University, MMC 11200 SW 8th Street (CP-304), Miami, Florida 33199, United States

*E-mail: tharrop@uga.edu

Table of Contents:

Experimental **S3-S7**

X-ray Crystallographic Data **S8-14**
- **Table S1.** Summary of Crystal Data/Intensity Collection for **2** and **3**•Et_2O **S9**
- **Table S2.** Selected bond distances and bond angles for **2** **S10**
- **Table S3.** Selected bond distances and bond angles for **3**•Et_2O **S11-13**
- **Figure S1.** Asymmetric unit of **2** **S14**
- **Figure S2.** Asymmetric unit of **3**•0.5Et_2O **S14**

Spectroscopic Data **S15-37**
- **X-ray absorption – general** **S15-16**
- **Figure S3.** XANES spectrum and EXAFS data for **1** **S16**
- **Table S4.** Summary of best-fit simulations to Ni EXAFS for **1** **S16**
- **Figure S4.** FTIR of **2** and **2-^{15}NO** **S17**
- **Figure S5.** Zoom-in of the FTIR of **2** and **2-^{15}NO** **S18**
- **Figure S6.** ^{1}H NMR spectrum of **2** **S19**
- **Figure S7.** ^{15}N NMR spectrum of **2-^{15}NO** **S20**
- **Figure S8.** HR-ESI-MS(-) of **2** **S21**
- **Figure S9.** Zoom-in of the HR-ESI-MS(-) of **2** **S22**
- **Figure S10.** HR-ESI-MS(-) of **2-^{15}NO** **S23**

Figure S11. Zoom-in of the HR-ESI-MS(-) of **2-^{15}NO** **S24**
Figure S12. FTIR of **3** and **3-^{15}NO** **S25**
Figure S13. ^{1}H NMR spectrum of **3** **S26**
Figure S14. ^{15}N NMR spectrum of **3-^{15}NO** **S27**
Figure S15. HR-ESI-MS(-) of **3** **S28**
Figure S16. Zoom-in of the HR-ESI-MS(-) of **3** **S29**
Figure S17. HR-ESI-MS(-) of **3-^{15}NO** **S30**
Figure S18. Zoom-in of the HR-ESI-MS(-) of **3-^{15}NO** **S31**
Figure S19. ^{1}H NMR of the Et_2O-soluble from workup of **2** compared to $nmpS_2$ **S32**
Figure S20. Zoom-in of the ^{1}H NMR from Figure S19 **S33**
Figure S21. ^{1}H NMR of the reaction of **2** with [Co(T(-OMe)PP)] **S34**
Figure S22. ^{1}H NMR from Fig. S21 compared to authentic Co-P and Co-P-NO **S35**
Figure S23. FTIR of the reaction of **2** and **2-^{15}NO** with [Co(T(-OMe)PP)] **S36**
Figure S24. ^{1}H NMR/IR of the free NO(g) transfer from **2**/**2-^{15}NO** to Co-P **S37**

Computational details **S38-45**
Table S5. Löwdin population analysis for selected MOs of **2*** **S39**
Table S6. Löwdin population analysis for selected MOs of **3*** **S40**
Table S7. Optimized BP86/def2-TZVPP Cartesian coordinates (Å) for **2*** **S41**
Table S8. Optimized BP86/def2-TZVPP Cartesian coordinates (Å) for **3*** **S42**
Figure S25. MO diagram of **2*** **S43**
Table S9. Bond Distances/Angles of **3** (X-ray) compared DFT-optimized **3*** **S44**
Figure S26. MO diagram of **3*** **S45**

References **S46-47**

Experimental

General Information. All reagents were purchased from commercial sources and used as received unless otherwise noted. Research grade nitric oxide gas (NO(g), UHP, 99.5%) was obtained from Matheson Tri-Gas that was purified by passage through an Ascarite II® column (sodium hydroxide-coated silica, purchased from Aldrich) and handled under anaerobic conditions. ^{15}NO(g) ($^{15}N \geq 98\%$) was procured from Cambridge Isotope Labs and used as received. Acetonitrile (MeCN), dichloromethane (CH_2Cl_2), tetrahydrofuran (THF), and diethyl ether (Et_2O) were purified by passage through activated alumina columns of an MBraun MB-SPS solvent purification system and stored under an N_2 atmosphere until use. *N*,*N*-dimethylformamide (DMF) was purified with a VAC solvent purifier containing 4 Å molecular sieves and stored under N_2. Methanol (MeOH) was stored over 3 Å molecular sieves for at least one week and degassed using the freeze-pump-thaw method. The synthesis of (Et_4N)[Ni(nmp)(SPh-*o*-NH_2-*p*-CF_3)] (**1**) is published elsewhere.[1] All reactions were performed under an N_2 atmosphere at room temperature (RT) using Schlenk-line techniques or under an atmosphere of purified N_2 in an MBraun Unilab glovebox. To minimize potential photoreactions, all reactions with NO(g) or Ni-nitrosyls were performed with minimal exposure to light (laboratory lights off) with reaction flasks wrapped in aluminum foil.

Physical Methods. Fourier transform infrared (FTIR) spectra were collected on a ThermoNicolet 6700 spectrophotometer running the OMNIC software. All FTIR samples were prepared in the glovebox. UV-vis spectra were collected at 25 °C using a Cary-50 spectrophotometer containing a Quantum Northwest TC 125 temperature control unit. All UV-vis samples were prepared in gastight Teflon-lined screw-cap quartz cells with an optical pathlength of 1 cm. ^1H NMR spectra were recorded in the listed deuterated solvent on either a 400 MHz Bruker BZH 400/52 NMR spectrometer or a Varian Unity Inova 500 MHz NMR spectrometer at RT with chemical shifts internally referenced to tetramethylsilane (TMS = $Si(CH_3)_4$), or the residual protio signal of the deuterated solvent as reported.[2] ^{15}N NMR spectra were recorded on the 500 MHz spectrometer and externally referenced to CH_3NO_2 ($\delta = 0.00$ ppm). Low-resolution electrospray ionization mass spectrometry (LR-ESI-MS) were collected using a Bruker Esquire 3000 plus ion trap mass spectrometer. High-resolution (HR-ESI-MS) were collected using an Orbitrap Elite system with precision to the third decimal place. Elemental analysis for C, H, and N was performed by QTI Intertek in Whitehouse, NJ.

Synthesis and Characterization of Compounds:

(Et_4N)$_2$[{Ni(κ^2-*S*Ph-*o*-*N*NO-*p*-CF_3)(NO)}$_2$] (2). NO(g) was purged into a red-brown DMF solution (3 mL) of **1** (0.0964 g, 0.1717 mmol) for 1 min. The solution changed color from red-brown to green-brown within 5-10 min and was primarily dark-green after ~15 min. The solution was stirred under NO(g) in the headspace of the flask for an additional 30 min. The DMF was then removed via short-path vacuum distillation and the resulting green residue was stirred with 3 × 5 mL portions of Et_2O that were decanted from the solid and collected. An additional 12 mL of Et_2O was added to the green solid that was stirred for 15 h. The green solid was collected via vacuum filtration (Et_2O filtrate combined with previous Et_2O washes) in a glass frit and washed with Et_2O (2 mL) then pentane (2 mL) to afford the $\{NiNO\}^{10}$ complex **2**. The Et_2O-soluble component was concentrated to afford 0.0162 g of a yellow oil that was identified by ^1H NMR to be primarily the

disulfide of nmp^{2-} (0.0447 mmol, 52% yield; see Figures S19-20). To avoid loss of coordinated NO, the solid was only dried for 5-10 min to yield 0.0866 g of compound that still contained nmpS$_2$. Based on ^{1}H NMR integration (see Figure S6), 0.0520 g of the total mass was from **2** (0.0592 mmol, 69%). Elemental analysis of vacuum-dried complex suggests loss of NO (see below). FTIR (KBr pellet) ν_{max} (cm^{-1}): 3447 (w), 3385 (w), 3214 (w), 3061 (w), 2985 (m), 2595 (vw), 2297 (w), 1871 (w), 1759 (vs, ν_{NO}), 1743 (vs, ν_{NO}), 1671 (m), 1633 (s), 1590 (s), 1563 (m), 1523 (m), 1475 (m), 1461 (m), 1409 (m), 1395 (m), 1375 (m), 1342 (m, ν_{NO}), 1324 (s), 1300 (m), 1258 (s, ν_{NN}), 1231 (s), 1209 (s), 1183 (s), 1164 (s), 1133 (m), 1100 (s), 1076 (s), 1032 (m), 1001 (m), 979 (m), 879 (m), 817 (m), 800 (m), 760 (m), 733 (w), 712 (m), 700 (w), 677 (m), 655 (w), 642 (w), 620 (w), 573 (w), 535 (vw), 493 (vw), 460 (w), 438 (vw), 421 (vw), 410 (vw). FTIR (DMSO, CaF$_2$ plates, 0.1 mm Teflon spacers) ν_{max} (cm^{-1}): 1784 (vs, ν_{NO}). ^{1}H NMR (500 MHz, CD$_3$CN, δ from protio solvent; nmpS$_2$ and other species present, only listing peaks assigned to **2**): 7.84 (s, 1H), 7.16 (d, 1H, *J* = 8.2 Hz), 6.88 (m, 1H, *J* = 8.2 Hz), 3.16 (q, 19H, *J* = 7.3 Hz), 1.19 (t, 27H, *J* = 7.3 Hz). UV-vis (DMF, 298 K) λ_{max}, nm (ε, M^{-1} cm^{-1}): 425 nm (3000), 686 nm (150). HR-ESI-MS (*m*/*z*): calc. for [M-2Et$_4$N]$^{2-}$, $C_7H_3F_3N_3NiO_2S$, 307.925 (100.0), 309.921 (43.0), 308.929 (7.6), 311.918 (7.0), 310.924 (2.9); found: 307.926 (100.0), 309.921 (42.3), 308.928 (7.1), 311.917 (6.6), 310.924 (2.5). Anal. calcd for $C_{30}H_{46}F_6N_5Ni_2OS_2$ (M-2NO): C, 44.04; H, 5.67; N, 10.27; Found: C, 44.02; H, 6.07; N, 9.36.

(Et$_4$N)$_2$[{Ni(κ^2-*S*Ph-*o*-*N*^{15}NO-*p*-CF$_3$)(^{15}NO)}$_2$] (2-^{15}NO). The reaction of **1** with ^{15}NO(g) was carried out as described above except for using 0.1066 g (0.1899 mmol) of **1** and ^{15}NO(g) to yield 0.0120 g of yellow oil (Et$_2$O-soluble) and 0.0625 g (0.0708 mmol, 75%) of **2-^{15}NO** as a green powder. All spectroscopic characterization was the same as for **2** except the following: FTIR (KBr pellet) ν_{max} (cm^{-1}): 1724 (vs, ν_{NO}), 1708 (vs, ν_{NO}), $\Delta\nu_{NO}$ from **2** = 35 cm^{-1}; 1326 cm^{-1} (s, ν_{NO}), $\Delta\nu_{NO}$ from **2** = 16 cm^{-1}; 1249 cm^{-1} (m, $\Delta\nu_{NN}$), $\Delta\nu_{NN}$ from **2** = 9 cm^{-1}. FTIR (DMSO, CaF$_2$ plates, 0.1 mm Teflon spacers) ν_{max} (cm^{-1}): 1747 (vs, ν_{NO}), $\Delta\nu_{NO}$ from **2** = 37 cm^{-1}. ^{15}N NMR (50.69 MHz, CD$_3$CN, δ from CH$_3$NO$_2$): 189.93, 108.74, 88.53, 39.02. HR-ESI-MS (*m*/*z*): calc. for [M-2Et$_4$N]$^{2-}$, $C_7H_3F_3{}^{15}N_2NNiO_2S$, 309.919 (100.0), 311.915 (43.0), 310.923 (7.6), 313.912 (7.0), 312.918 (2.9); found: 309.920 (100.0), 311.915 (41.5), 310.922 (6.9), 313.911 (6.5), 312.918 (2.3).

***trans*-(Et$_4$N)$_2$[Ni(κ^2-*S*Ph-*o*-*N*NO-*p*-CF$_3$)$_2$] (3).** A saturated MeCN solution (1 mL) of **2** (0.0100 g, 0.0114 mmol) was slowly (months) diffused with Et$_2$O at -25 °C to yield red crystals of **3** (0.0010 g, 0.0013 mmol, 11%). The mother-liquor was removed, the remaining solid stirred with Et$_2$O (5 × 4 mL) that was decanted, and the crystals were dried on high vac. FTIR (KBr pellet) ν_{max} (cm^{-1}): 3444 (vw), 2982 (w), 1760 (m, ν_{NO}), 1743 (m, ν_{NO}), 1587 (w), 1562 (vw), 1481 (m), 1460 (m), 1413 (m), 1397 (m), 1361 (m), 1339 (m, ν_{NO}), 1323 (vs), 1300 (m), 1258 (s, ν_{NN}), 1230 (s), 1207 (m), 1161 (s), 1101 (vs), 1077 (vs), 1031 (m), 979 (w), 879 (vw), 800 (s), 712 (w), 677 (w), 655 (vw), 622 (vw), 572 (vw), 495 (vw), 478 (vw), 461 (vw), 416 (vw). ^{1}H NMR (500 MHz, CD$_3$CN, δ from protio solvent): 7.50 (d, 2H, *J* = 1.2 Hz), 7.15 (d, 2H, *J* = 8.1Hz), 6.91 (m, 2H), 3.16 (q, 20H, *J* = 7.3), 1.20 (t, 28H, *J* = 7.3 Hz). HR-ESI-MS (*m*/z): calc. for [M-2Et$_4$N]$^{2-}$, $C_{14}H_6F_6N_4NiO_2S_2$, 248.960 (100.0), 249.462 (15.1), 249.958 (47.5), 250.459 (5.8), 250.956 (4.9); found: 248.960 (100.0), 249.461 (16.6), 249.957 (48.3), 250.459 (7.9), 250.956 (9.0).

***trans*-(Et$_4$N)$_2$[Ni(κ^2-*S*Ph-*o*-*N*^{15}NO-*p*-CF$_3$)$_2$] (3-^{15}NO).** This reaction was carried out as described above except for using **2-^{15}NO** (0.0100 g, 0.0113 mmol) and isolating 0.0024 g (0.0032 mmol, 28%) of **3-^{15}NO**. All spectroscopic characterization was the same as for **2** except the following:

FTIR (KBr pellet) ν_{max} (cm^{-1}): 1324 (m, ν_{NO}), $\Delta\nu_{NO}$ from **3** = 15 cm^{-1}; 1250 (s, ν_{NN}), $\Delta\nu_{NN}$ from **3** = 8 cm^{-1}. ^{15}N NMR (50.69 MHz, CD_3CN, δ from CH_3NO_2), 193.98. HR-ESI-MS (m/z): calc. for $[M\text{-}2Et_4N]^{2-}$, $C_{14}H_6F_6N_2{}^{15}N_2NiO_2S_2$, 249.957 (100.0), 250.459 (15.1), 250.955 (47.5), 251.456 (5.8), 251.953 (3.5); found: 249.957 (100.0), 250.458 (15.1), 250.953 (47.6), 251.456 (7.8), 251.953 (9.1).

Nmp-disulfide (nmpS_2). Picolyl chloride was generated *in situ* by refluxing picolinic acid (1.3406 g, 10.889 mmol) in 10 mL of $SOCl_2$ for 2 h to yield a dark-purple mixture. After vacuum distilling the $SOCl_2$ and triturating with CH_2Cl_2 (7 mL), the solid was dissolved in 10 mL of CH_2Cl_2 to yield a dark-blue heterogeneous mixture. After 15 min of stirring at 0 °C, a CH_2Cl_2 solution (10 mL) containing cystamine dihydrochloride (1.0315 g, 4.5804 mmol) and Et_3N (6.5 mL, 47 mmol) was added. The mixture was stirred at RT under N_2 for 4 d, which was then partitioned between 200 mL of CH_2Cl_2 and 200 mL of DI H_2O. Solid K_2CO_3 was added to the aqueous layer until it remained basic (fizzing stopped), which changed the CH_2Cl_2 solution color from dark-blue to purple-blue. The organic layer was extracted and washed with saturated $NaHCO_3$ solution (2 × 200 mL) and brine (2 × 200 mL), then dried over $MgSO_4$, filtered, and concentrated to a brown oil. After a failed chromatographic purification with silica, the crude product was redissolved in 50 mL of CH_2Cl_2 and washed with saturated $NaHCO_3$ solution (50 mL) and brine (3 × 50 mL), then dried over $MgSO_4$. After filtering off the $MgSO_4$, the organic phase was concentrated to a yellow oil and ~2 mL of CH_2Cl_2 was added followed by 40 mL of hexane. Rigorous stirring of this solvent mixture resulted in a white powder that was collected via vacuum filtration and dried on a high vacuum line overnight to afford 0.6658 g (1.837 mmol, 40%) of product. FTIR (KBr pellet) ν_{max} (cm^{-1}): 3371 (m, ν_{NH}), 3355 (m, ν_{NH}), 3060 (vw), 3013 (vw), 2979 (vw), 2952 (vw), 2917 (w), 2861 (vw), 1660 (vs, ν_{CO}), 1619 (w), 1591 (m), 1569 (m), 1521 (vs), 1467 (m), 1433 (m), 1420 (w), 1353 (vw), 1307 (vw), 1286 (m), 1245 (w), 1222 (w), 1190 (vw), 1182 (vw), 1164 (w), 1148 (w), 1090 (w), 1041 (w), 999 (m), 904 (vw), 867 (vw), 853 (vw), 828 (vw), 818 (w), 749 (m), 724 (vw), 702 (w), 692 (w), 639 (m), 619 (m), 515 (vw). ^1H NMR (500 MHz, $CDCl_3$, δ from protio solvent): 8.55 (d, 2H, J = 4.8 Hz), 8.41 (br s, 2H, N*H*), 8.18 (d, 2H, J = 7.8 Hz), 7.84 (td, 2H, J = 7.7 Hz, 1.7 Hz), 7.42 (m, 2H), 3.82 (q, 4H, J = 6.3 Hz), 2.96 (t, 4H, J = 6.4 Hz). ^{13}C NMR (100 MHz, $CDCl_3$, δ from solvent signal): 164.61 (*C*=O), 149.80, 148.29, 137.48, 126.39, 122.35, 38.27, 37.99. LR-ESI-MS (*m*/*z*): calc. for $[M+H]^+$, $C_{16}H_{19}N_4O_2S_2$, 363.1 (100.0), 364.1 (20.4), 365.1 (10.4). Found: 363.1 (100.0), 364.1 (33.2), 365.1 (11.3); calc. for $[M+Na]^+$, $C_{16}H_{18}N_4O_2S_2Na$, 385.1 (100.0), 386.1 (20.4), 387.1 (10.4). Found: 385.1 (100.0), 386.1 (18.8), 387.1 (10.7); calc. for $[2M+Na]^+$, $C_{32}H_{36}N_8O_4S_4Na$, 747.2 (100.0), 748.2 (40.8), 749.2 (25.0), 750.2 (6.3), 751.2 (2.2). Found: 747.0 (100.0), 748.0 (38.8), 749.0 (25.7), 750.0 (8.3), 751.0 (4.3).

Reactivity:

Reaction of 1^{ox} with NO(g). To a 4 mL DMF solution of **1** (0.0398 g, 0.0709 mmol) was added ceric ammonium nitrate (0.0385 g, 0.0702 mmol). The resulting dark green solution was then purged with a stream of NO(g) for 1 min at RT, and the solution immediately changed color to red-brown. Red insoluble precipitate was observed almost immediately. The mixture was stirred for 1 h under an NO(g) atmosphere, and the DMF was removed by short-path vacuum distillation. The resulting red-brown residue was stirred in Et_2O overnight and filtered to afford 0.0562 g of a red-brown product. This product is sparingly soluble in all organic solvents attempted (DMF, MeCN, CH_2Cl_2). FTIR analysis reveals this product to be the tetrameric complex $[Ni_4(nmp)]_4$. FTIR (KBr pellet) ν_{max} (cm^{-1}): 3239 (w, br), 3084 (w, br), 2937 (w), 1748 (w), 1653 (ν_{CO}, vs), 1598 (m), 1438 (s), 1382 (s) 1325 (s), 1132 (m), 1034 (m), 821 (m), 734 (m), 675 (s), 623 (w).

UV-vis monitor of 1 with $NOBF_4$. To a 5 mM DMF stock of **1** was added one equiv of $NOBF_4$, which resulted in immediate bleaching of the solution and an absence of any spectral features in the UV-vis absorbance profile. This bleaching, in addition to formation of a red precipitate (verified by IR and NMR), is consistent with formation of $[Ni(nmp)]_4$ and RSSR.

Reaction of 2 with [Fe(TPP)Cl]. To a 2 mL MeCN solution containing 0.0183 g (0.0260 mmol) of [Fe(TPP)Cl] was added **2** (0.0115 g, 0.0131 mmol) in 2 mL of MeCN. There was no observed color change. The dark red-brown solution was then stirred for 2 h in the dark at RT and the MeCN was removed in vacuo. The brown residue was stirred in 6 mL of MeOH and filtered to afford 0.0164 g of a purple solid. FTIR analysis shows that the purple solid is unreacted [Fe(TPP)Cl] (0.02346 mmol, 90 % recovery).

NO Transfer from 2 to [Co(T-(OMe)PP)]. To a 2 mL CH_2Cl_2 solution containing 0.0158 g (0.0200 mmol) of [Co(T-(OMe)PP)] was added **2** (0.0087 g, 0.0099 mmol) in 1.5 mL of CH_2Cl_2 with no observed color change. The red-brown solution was stirred for 24 h in the dark at RT, and the CH_2Cl_2 was removed in vacuo. The brown residue was stirred in 6 mL of MeOH and filtered to afford 0.0156 g of a purple solid (MeOH-insoluble). FTIR analysis shows that the purple solid contains the $\{CoNO\}^8$ complex (ν_{NO} = 1694 cm^{-1} in KBr, Fig. S23), [Co(T-(OMe)PP)(NO)]. Quantification was done by integrating peaks corresponding to [Co(T(-OMe)PP)] and [Co(T(-OMe)PP)(NO)] in the ^1H NMR in CD_2Cl_2 (see Figs. S21-22).

^{15}NO Transfer from 2-^{15}NO to [Co(T-(OMe)PP)]. This reaction was performed under identical conditions as with **2** except for using 0.0093 g (0.011 mmol) of **2-^{15}NO** and 0.0167 g (0.0211 mmol) of [Co(T-(OMe)PP)] to afford 0.0161 g of a purple solid. FTIR analysis shows that the purple solid contains the $\{CoNO\}^8$ complex [Co(T-(OMe)PP)(^{15}NO)] (ν_{NO} = 1663 cm^{-1} in KBr, see Fig. S23; $\Delta\nu_{NO}$ from [Co(T-(OMe)PP)(NO)] = 31 cm^{-1}).

Vial-vial NO(g) Transfer from 2/2-^{15}NO to [Co(T-(OMe)PP)]. A 1-dram vial was charged with 13.7 mg (0.0155 mmol) of **2-^{15}NO**. This vial was then placed into a larger (5-dram) vial containing 3 mL of a 20 mM CH_2Cl_2 solution of [Co(T-(OMe)PP)] (0.060 mmol per vial) and the entire reaction vessel was sealed with a white rubber septum using wire and electrical tape to ensure a proper seal. To the inner vial containing solid **2-^{15}NO** was then added 1 mL of MeCN through the septum. The reaction vessel was kept sealed for 24 h in the dark at RT prior to workup (removing

the CH_2Cl_2 in the outer vial by vacuum) and spectroscopic analysis (^{1}H NMR in CD_2Cl_2 and FTIR, see Fig. S24 on pg. S37). Notably, the initial green color of the $\{NiNO\}^{10}$ MeCN solution in the inner (1-dram) vial changed to red over the 24 h reaction time and ESI-MS(-) confirmed the presence of **3-^{15}NO**. Based on ^{1}H NMR integration, 43% of the dissolved Co(II)-P (0.060 mmol) was converted into the Co-nitrosyl (0.026 mmol), overall yield = 81% (0.032 mmol possible). The same setup was used with **2** except for using 18.1 mg of **2** (0.0206 mmol).

Structural data:

X-ray Crystallographic Data Collection and Structure Solution and Refinement. Green blade crystals of **2** were grown by vapor diffusion of Et_2O into a MeCN solution of **2** at -25 °C. A dark green crystal was mounted on the top of a glass fiber. The X-ray intensity data were measured at 100 K on a Bruker SMART APEX II X-ray diffractometer system with graphite-monochromated Mo Kα radiation (λ = 0.71073 Å) using the ω-scan technique. The data were collected in 1464 frames with 10 s exposure times. The data were corrected for Lorentz and polarization effects[3] and integrated with the manufacturer's SAINT software. Absorption corrections were applied with the program SADABS. Subsequent structure refinement was performed using the SHELXTL-2013[4, 5] solution package operating on a Pentium computer. The structure was solved by direct methods using the SHELXTL-2013 software package. Non-hydrogen atomic scattering factors were taken from the literature tabulations.[6] Non-hydrogen atoms were located from successive difference Fourier map calculations. Dark-red crystals of **3** were obtained by vapor diffusion of Et_2O into a saturated solution of **2** in MeCN at -25 °C for 4 months. The crystal was mounted on the tip of a glass fiber. The X-ray intensity data were measured at 100 K on a Bruker D8 Quest PHOTON 100 CMOS X-ray diffractometer system with Incoatec Microfocus Source (IμS) monochromated Mo Kα radiation (λ = 0.71073 Å) using the ϕ and ω-scan technique. The data were collected in 2200 frames with 10 s exposure times. The data were corrected for Lorentz and polarization effects and integrated with the manufacturer's SAINT software. Absorption corrections were applied with TWINABS.

For **2**, the atoms of one of the NO groups and the CF_3 group in the molecule were found disordered in the adjacent positions in two sets of each. For NO: N(2), O(1) (one set; 65% occupancies) and N(2′), O(1′) (another set; 35% occupancies). For fluorine: F(1), F(2), F(3) (one set; 51% occupancies) and F(1′), F(2′), F(3′) (another set; 49% occupancies). Each of these two sets is divided using the PART commands and proper restraints. In the final cycles of each refinement, all non-hydrogen atoms were refined in anisotropic displacement parameters. Selected data and metric parameters for **2** and **3** are summarized in Tables S1-S3. Perspective views of the complexes were obtained using ORTEP,[7] omitting N(2′), O(1′), F(1′), F(2′), F(3′), and hydrogen atoms for clarity.

For **3**, the F atoms from each of three CF_3 groups of the ligands in the two crystallographically distinct molecules were found disordered in the adjacent positions in two sets of each. The disordered atoms are labeled as follows with refined occupancies for each set: (i) for fluorine atoms: F(1), F(2), F(3) (one set; 82% occupancies) and F(1′), F(2′), F(3′) (another set; 18% occupancies); (ii) for fluorine atoms: F(7), F(8), F(9) (one set; 63% occupancies) and F(7′), F(8′), F(9′) (another set; 37% occupancies); (iii) for fluorine atoms: F(10), F(11), F(12) (one set; 67% occupancies) and F(10′), F(11′), F(12′) (another set; 33% occupancies). Each of these two sets is divided using the PART commands and proper restraints. Moreover, the carbon atoms C(53), C(54), C(55), C(56), C(57), C(58), C(59), C(60), C(61), C(62), C(63), C(64) from two halves of the Et_4N^+ cations were found disordered around their related symmetries of an inversion center. While the twinning feature was detected, the reflections were indexed in two domains using CELL_NOW. While the HKLF4 format file was used for structure solution, the HKLF5 format file was used for refinement. In the final cycles of each refinement, all the non-hydrogen atoms were refined in anisotropic displacement parameters. Perspective views of the complexes were obtained using ORTEP,[7] omitting N(2′), O(1′), F(1′), F(2′), F(3′), and hydrogen atoms for clarity.

Table S1. Summary of Crystal Data and Intensity Collection and Structure Refinement Parameters for $(Et_4N)_2[\{Ni(\kappa^2\text{-}S\text{Ph-}o\text{-}N\text{NO-}p\text{-}CF_3)(NO)\}_2]$ (**2**) and *trans*-$(Et_4N)_2[Ni(\kappa^2\text{-}S\text{Ph-}o\text{-}N\text{NO-}p\text{-}CF_3)_2]\bullet 0.5Et_2O$ (**3**•$0.5Et_2O$).

**Parameters**	**2**	**3**•$0.5Et_2O$
Formula	$C_{30}H_{46}F_6N_8Ni_2O_4S_2$	$C_{63}H_{88}F_{12}N_{12}Ni_2O_5S_4$
Formula weight	878.28	1567.11
Crystal system	Monoclinic	Triclinic
Space group	*C* 2/c	*P*1
Crystal color, habit	Green, square	Dark red, plate
a, Å	23.2608(14)	13.3985(6)
b, Å	10.7528(6)	16.8518(9)
c, Å	17.4230(10)	18.0347(9)
α, deg	90	112.816(1)
β, deg	117.9760(10)	92.719(1)
γ, deg	90	91.167(2)
V, $Å^3$	3848.6(4)	3745.9(3)
Z	4	2
ρ_{calcd}, g/cm^3	1.516	1.389
T, K	100(2)	100(2)
abs coeff, μ (Mo Kα), mm^{-1}	1.161	0.697
θ limits, deg	1.983-30.500	1.968-26.458
total no. of data	30284	15409
no. of unique data	5892	15409
no. of parameters	282	908
GOF of F^2	1.054	1.028
R_1,[a] %	0.0468	0.0745
wR_2,[b] %	0.1463	0.2002
max, min peaks, e/$Å^3$	1.426, -0.578	2.254, -0.922

$^aR_1 = \Sigma| |F_o| - |F_c| | / \Sigma |F_o|$; $^bwR_2 = \{\Sigma[w(F_o^2 - F_c^2)^2]/\Sigma[w(F_o^2)^2]\}^{1/2}$

Table S2. Selected bond distances (Å) and bond angles (deg) for $(Et_4N)_2$[{Ni(κ[2]-*S*Ph-*o*-*N*NO-*p*-CF_3)(NO)}$_2$] (**2**).

Ni(1)-N(2')	1.621(11)	C(1)-S(1)-Ni(1)	96.14(8)
Ni(1)-N(2)	1.659(7)	C(1)-S(1)-Ni(1)#1	102.68(8)
Ni(1)-N(1)	1.971(2)	Ni(1)-S(1)-Ni(1)#1	82.07(2)
Ni(1)-S(1)	2.3169(7)	N(3)-N(1)-C(2)	113.5(2)
Ni(1)-S(1)#1	2.3555(7)	N(3)-N(1)-Ni(1)	126.90(17)
S(1)-C(1)	1.767(3)	C(2)-N(1)-Ni(1)	119.63(15)
S(1)-Ni(1)#1	2.3555(7)	O(1)-N(2)-Ni(1)	167.8(12)
N(1)-N(3)	1.299(3)	O(1')-N(2')-Ni(1)	170(2)
N(1)-C(2)	1.422(3)	C(2)-C(1)-C(6)	118.5(2)
N(2)-O(1)	1.182(8)	C(2)-C(1)-S(1)	120.53(19)
N(2')-O(1')	1.162(12)	C(6)-C(1)-S(1)	120.9(2)
C(1)-C(2)	1.409(3)	C(3)-C(2)-C(1)	119.9(2)
C(1)-C(6)	1.401(4)	C(3)-C(2)-N(1)	124.1(2)
C(2)-C(3)	1.386(4)	C(1)-C(2)-N(1)	116.0(2)
C(3)-C(4)	1.392(4)	C(2)-C(3)-C(4)	120.3(3)
C(4)-C(5)	1.387(5)	C(3)-C(4)-C(5)	120.3(3)
C(4)-C(7)	1.486(5)	C(3)-C(4)-C(7)	119.5(3)
C(5)-C(6)	1.374(4)	C(5)-C(4)-C(7)	120.1(3)
C(7)-F(1')	1.307(13)	C(6)-C(5)-C(4)	119.7(3)
C(7)-F(2)	1.258(18)	C(5)-C(6)-C(1)	121.3(3)
C(7)-F(3)	1.330(8)	F(2)-C(7)-F(3)	112.9(12)
C(7)-F(2')	1.405(16)	F(1')-C(7)-F(2')	102.7(10)
C(7)-F(3')	1.408(12)	F(1')-C(7)-F(3')	110.2(8)
C(7)-F(1)	1.429(15)	F(2')-C(7)-F(3')	102.9(11)
N(4)-C(9)	1.499(4)	F(1')-C(7)-C(4)	117.9(10)
N(4)-C(11)	1.503(4)	F(2)-C(7)-C(4)	115.7(11)
N(4)-C(15)	1.527(4)	F(3)-C(7)-C(4)	113.8(4)
N(4)-C(13)	1.551(4)	F(2')-C(7)-C(4)	111.7(10)
C(8)-C(9)	1.510(5)	F(3')-C(7)-C(4)	110.2(5)
C(10)-C(11)	1.511(5)	F(2)-C(7)-F(1)	110.8(11)
C(12)-C(13)	1.503(4)	F(3)-C(7)-F(1)	97.1(8)
C(14)-C(15)	1.545(4)	C(4)-C(7)-F(1)	104.5(8)
N(3)-O(2)	1.269(3)	C(9)-N(4)-C(11)	110.4(2)
		C(9)-N(4)-C(15)	113.1(2)
N(2')-Ni(1)-N(1)	126.0(12)	C(11)-N(4)-C(15)	109.5(2)
N(2)-Ni(1)-N(1)	133.6(5)	C(9)-N(4)-C(13)	107.6(2)
N(2')-Ni(1)-S(1)	118.3(10)	C(11)-N(4)-C(13)	110.0(2)
N(2)-Ni(1)-S(1)	123.8(5)	C(15)-N(4)-C(13)	106.1(2)
N(1)-Ni(1)-S(1)	86.21(6)	N(4)-C(9)-C(8)	116.6(3)
N(2')-Ni(1)-S(1)#1	122.0(13)	N(4)-C(11)-C(10)	116.5(3)
N(2)-Ni(1)-S(1)#1	109.8(6)	C(12)-C(13)-N(4)	114.5(2)
N(1)-Ni(1)-S(1)#1	99.10(6)	N(4)-C(15)-C(14)	113.3(2)
S(1)-Ni(1)-S(1)#1	96.66(2)	O(2)-N(3)-N(1)	114.2(2)

Table S3. Selected bond distances (Å) and bond angles (deg) for *trans*-$(Et_4N)_2$[Ni(κ^2-*S*Ph-*o*-*N*NO-*p*-$CF_3)_2$]•0.5Et_2O (**3**•0.5Et_2O).

Ni1—S1	2.2211 (16)	N6—C28	1.502 (7)
Ni1—S2	2.1991 (16)	N6—C30	1.512 (7)
Ni1—N1	1.885 (5)	C23—C24	1.509 (8)
Ni1—N3	1.899 (5)	C25—C26	1.511 (8)
S1—C1	1.754 (6)	C27—C28	1.513 (8)
S2—C8	1.753 (6)	C29—C30	1.515 (9)
N1—N2	1.309 (7)	Ni2—S3	2.2044 (16)
N1—C2	1.426 (7)	Ni2—S4	2.2042 (17)
N2—O1	1.279 (6)	Ni2—N7	1.898 (5)
N3—N4	1.317 (7)	Ni2—N9	1.903 (5)
N3—C9	1.426 (7)	S3—C31	1.757 (6)
N4—O2	1.251 (6)	S4—C38	1.752 (6)
C1—C2	1.409 (8)	N7—N8	1.312 (7)
C1—C6	1.386 (8)	N7—C32	1.416 (7)
C2—C3	1.390 (8)	N8—O3	1.262 (6)
C3—C4	1.393 (9)	N9—N10	1.297 (7)
C4—C5	1.384 (9)	N9—C39	1.426 (7)
C4—C7	1.474 (9)	N10—O4	1.263 (6)
C5—C6	1.408 (9)	C31—C32	1.401 (8)
C7—F1	1.305 (8)	C31—C36	1.389 (8)
C7—F2	1.355 (10)	C32—C33	1.384 (8)
C7—F3	1.324 (8)	C33—C34	1.389 (9)
C8—C9	1.380 (9)	C34—C35	1.390 (9)
C8—C13	1.399 (9)	C34—C37	1.465 (9)
C9—C10	1.395 (8)	C35—C36	1.375 (9)
C10—C11	1.388 (8)	C37—F7	1.348 (13)
C11—C12	1.391 (9)	C37—F8	1.288 (12)
C11—C14	1.478 (9)	C37—F9	1.364 (12)
C12—C13	1.386 (9)	C38—C39	1.407 (8)
C14—F4	1.317 (8)	C38—C43	1.390 (9)
C14—F5	1.284 (8)	C39—C40	1.377 (8)
C14—F6	1.353 (8)	C40—C41	1.400 (9)
N5—C16	1.516 (7)	C41—C42	1.389 (10)
N5—C18	1.533 (7)	C41—C44	1.472 (10)
N5—C20	1.508 (7)	C42—C43	1.403 (10)
N5—C22	1.522 (7)	C44—F10	1.281 (11)
C15—C16	1.516 (8)	C44—F11	1.356 (10)
C17—C18	1.495 (8)	C44—F12	1.354 (10)
C19—C20	1.506 (8)	N11—C46	1.530 (7)
C21—C22	1.525 (9)	N11—C48	1.554 (7)
N6—C24	1.530 (7)	N11—C50	1.501 (7)
N6—C26	1.528 (7)	N11—C52	1.534 (7)

C45—C46	1.498 (8)
C47—C48	1.499 (9)
C49—C50	1.509 (8)
C51—C52	1.493 (8)
N12—C54	1.730 (12)
N12—C56	1.90 (2)
C53—C54	1.573 (18)
C55—C56	1.523 (17)
N13—C60	1.557 (12)
N13—C61	1.547 (14)
N13—C63	1.553 (13)
N13—C64	1.582 (15)
C59—C60	1.58 (3)
C61—C64	1.80 (3)
C62—C63	1.557 (16)
C63—C64	1.81 (3)
O5—C66	1.285 (13)
O5—C67	1.464 (15)
C65—C66	1.561 (15)
C67—C68	1.601 (16)
S2—Ni1—S1	164.30 (7)
N1—Ni1—S1	85.10 (15)
N1—Ni1—S2	93.39 (15)
N1—Ni1—N3	178.5 (2)
N3—Ni1—S1	94.89 (14)
N3—Ni1—S2	86.20 (14)
C1—S1—Ni1	94.1 (2)
C8—S2—Ni1	96.6 (2)
N2—N1—Ni1	132.4 (4)
N2—N1—C2	112.5 (5)
C2—N1—Ni1	115.2 (4)
O1—N2—N1	114.6 (4)
N4—N3—Ni1	129.3 (4)
N4—N3—C9	113.2 (5)
C9—N3—Ni1	117.5 (4)
O2—N4—N3	115.5 (5)
C2—C1—S1	116.8 (4)
C6—C1—S1	124.4 (5)
C6—C1—C2	118.8 (6)
C1—C2—N1	113.8 (5)
C3—C2—N1	124.8 (5)
C3—C2—C1	121.4 (5)
C2—C3—C4	119.4 (6)
C3—C4—C7	120.4 (6)
C5—C4—C3	119.7 (6)
C5—C4—C7	119.9 (6)
C4—C5—C6	121.1 (6)
C1—C6—C5	119.7 (6)
F1—C7—C4	114.1 (6)
F1—C7—F2	101.2 (7)
F1—C7—F3	109.5 (7)
F2—C7—C4	113.1 (6)
F3—C7—C4	114.4 (6)
F3—C7—F2	103.3 (7)
C9—C8—S2	117.6 (5)
C9—C8—C13	119.5 (6)
C13—C8—S2	122.9 (5)
C8—C9—N3	115.0 (5)
C8—C9—C10	120.8 (6)
C10—C9—N3	124.1 (5)
C11—C10—C9	119.2 (6)
C10—C11—C12	120.6 (6)
C10—C11—C14	119.1 (6)
C12—C11—C14	120.3 (6)
C13—C12—C11	119.7 (6)
C12—C13—C8	120.2 (6)
F4—C14—C11	115.0 (6)
F4—C14—F6	101.8 (6)
F5—C14—C11	115.4 (6)
F5—C14—F4	107.4 (6)
F5—C14—F6	105.1 (7)
F6—C14—C11	110.8 (6)
C16—N5—C18	111.6 (4)
C16—N5—C22	111.0 (4)
C20—N5—C16	106.9 (4)
C20—N5—C18	110.7 (4)
C20—N5—C22	111.7 (4)
C22—N5—C18	105.0 (4)
N5—C16—C15	115.5 (5)
C17—C18—N5	115.0 (5)
C19—C20—N5	115.8 (5)
N5—C22—C21	113.7 (5)
C26—N6—C24	111.7 (5)
C28—N6—C24	105.0 (4)
C28—N6—C26	112.7 (4)
C28—N6—C30	111.2 (5)
C30—N6—C24	111.2 (4)
C30—N6—C26	105.2 (4)
C23—C24—N6	114.8 (5)
C25—C26—N6	115.3 (5)
N6—C28—C27	115.3 (5)

C29—C30—N6	116.3 (5)
S4—Ni2—S3	165.76 (7)
N7—Ni2—S3	85.93 (15)
N7—Ni2—S4	93.88 (16)
N7—Ni2—N9	177.5 (2)
N9—Ni2—S3	93.73 (15)
N9—Ni2—S4	85.85 (15)
C31—S3—Ni2	96.2 (2)
C38—S4—Ni2	94.6 (2)
N8—N7—Ni2	130.2 (4)
N8—N7—C32	112.0 (5)
C32—N7—Ni2	117.7 (4)
O3—N8—N7	114.5 (5)
N10—N9—Ni2	130.8 (4)
N10—N9—C39	113.1 (5)
C39—N9—Ni2	116.0 (4)
O4—N10—N9	116.0 (5)
C32—C31—S3	117.3 (4)
C36—C31—S3	124.1 (5)
C36—C31—C32	118.6 (5)
C31—C32—N7	114.4 (5)
C33—C32—N7	125.1 (5)
C33—C32—C31	120.5 (5)

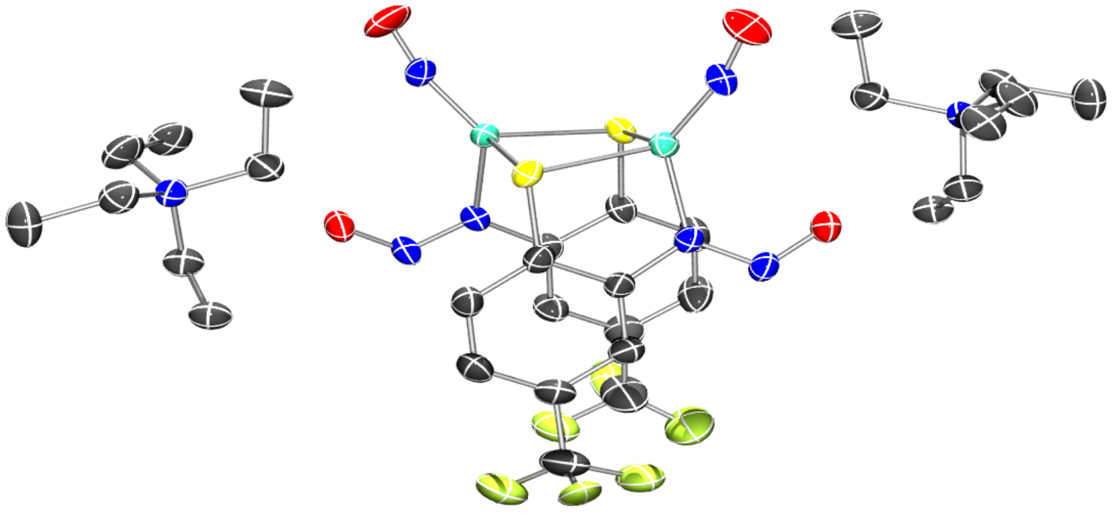

Figure S1. ORTEP of the asymmetric unit in **2** at 50% thermal probability ellipsoids. H atoms are omitted for clarity. Color scheme: Ni = aquamarine; S = yellow; N = blue; O = red; F = yellow-green; C = black.

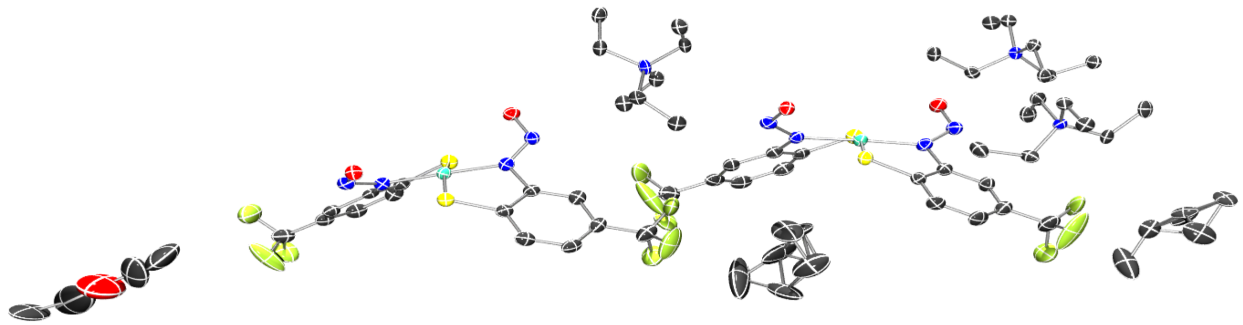

Figure S2. ORTEP of the asymmetric unit in **3**•0.5Et_2O at 50% thermal probability ellipsoids. H atoms are omitted for clarity. Color scheme: Ni = aquamarine; S = yellow; N = blue; O = red; F = yellow-green; C = black.

X-ray absorption spectroscopy (XAS) of 1. Nickel XAS data were collected on complex **1**. This complex is oxygen-sensitive and was prepared in an anaerobic glovebox immediately prior to data collection. Solid samples of **1** were diluted with inert BN at a 4:1 BN/sample ratio (w/w). XAS data were collected at the Stanford Synchrotron Radiation Lightsource (SSRL) on beamline 7-3, which utilizes a Si[220] double-crystal monochromator with an inline mirror for X-ray focusing and for harmonic rejection. During data collection, samples were maintained at 12 K using a liquid He continuous flow cryostat. Transmittance XAS spectra were collected for the solid compound. All spectra were measured in 5 eV increments in the pre-edge region (8265−8325 eV), 0.25 eV increments in the edge region (8325−8405 eV), and 0.05 $Å^{-1}$ increments in the extended X-ray absorption fine structure (EXAFS) region (out to k = 14 $Å^{-1}$), integrating from 1 to 25 s in a k3-weighted manner for a total scan length of approximately 50 min. X-ray energies were individually calibrated by collecting Ni-foil absorption spectra simultaneously with the compound spectra; the first inflection point of the Ni-foil spectrum was assigned at 8333 eV. Data represent the average of four to six scans. All spectra were collected on independent duplicate samples to ensure spectral reproducibility.

XAS spectra were processed using the Macintosh OS X version of the EXAFSPAK program suite[8] integrated with the Feff v8 software[9] for theoretical model generation. Data reduction utilized a Gaussian spline for background removal in the pre-edge region and a three-region cubic spline throughout the EXAFS. Data were converted to k space using a Ni E_0 value of 8333 eV. The k3-weighted EXAFS was truncated between 1.0 and 13.0 $Å^{-1}$ for filtering purposes. This k range corresponds to a spectral resolution of ca. 0.12 Å for all Ni−ligand interactions; therefore, only independent scattering environments outside 0.12 Å were considered resolvable in the EXAFS fitting analysis.[10] EXAFS fitting analysis was performed first on filtered data and then verified on the raw unfiltered data. EXAFS data were fit using both single- and multiple-scattering amplitude and phase functions calculated with the program Feff v8. Single-scattering theoretical models were calculated for carbon, nitrogen, and sulfur coordination to simulate Ni nearest-neighbor ligand environments. Scale factor (Sc) and E_0 values, used in a static manner during the simulations, were calibrated by fitting crystallographically characterized Ni models; specific values include a Sc = 0.9 and E_0 values of −10.55 eV for N/C and −12.22 eV for S. Criteria for judging the best-fit simulation utilized both the lowest mean square deviation between data and fit (F′), corrected for the number of degrees of freedom, and a reasonable Debye-Waller factor.[11, 12] Pre-edge analysis was done using EDG_FIT software. A spline function was best-fit between 8328 and 8342 eV, and a two-peak model was applied to accommodate the 1s→3d and 1s→4p pre-edge features.

X-ray Absorption Near Edge Spectral (XANES) analysis for **1** are consistent with nickel existing in the Ni(II) oxidation state, as indicated by the normalized half height of the rising edge at ca. 8340 eV.[13] Edge features, illustrated in Figure S3, showing the excitation jump max at ca. 8346 eV, is similar to that observed for the other $[Ni(nmp)(SR)]^-$ complexes as published earlier,[14] and suggests a ligand environment that is a mixture of both sulfur and nitrogen ligands. Analysis of the 1s→3d pre-edge transition in the XANES spectrum of **1** show two peaks one centered at ca. 8331.6 eV and the other at ca. 8334 eV. The two peaks likely represent the $1s\rightarrow3d_{x2-y2}$ and $1s\rightarrow3d_{z2}$ transitions, indicating possible axial ligation for the Ni ligand environment in the sample.[15] The corresponding 1s→3d peak area value for **1** is 1.84×10^{-2} eV^2 (range: 8330-8334 eV). In addition, a 1s→4p transition is also clearly observed for **1** centered at 8336.3 eV. The presence of prominent 1s→4p transitions is indicative of a square-planar geometry for **1**. The 1s→3d peak areas for this sample is within the range for planar complexes.[16]

Analysis of the Extended X-ray Absorption Fine Structure (EXAFS) spectra are consistent with the Ni coordination environment in **1** constructed of 2 O/N and 2 S ligands. Simulations of the Ni EXAFS for the samples (Figure S3) show a best fit with both O/N and S ligation in the nearest neighbor ligand coordination environment. The Ni in **1** is coordinated to two O/N ligands at a distance of 1.90 Å and two S ligands at a distance of 2.17 Å (Table S4). These distances match the average 4-coordinate square-planar N_2S_2 environments with average Ni-O/N and Ni-S distances of 1.93 Å and 2.18 Å, respectively, as listed in the Cambridge structural database.[17] Complex **1** has long-range scattering arising from multiple carbon atoms in the compound with slight differences in bond distances at 2.78 Å, 3.33 Å and 3.82 Å.

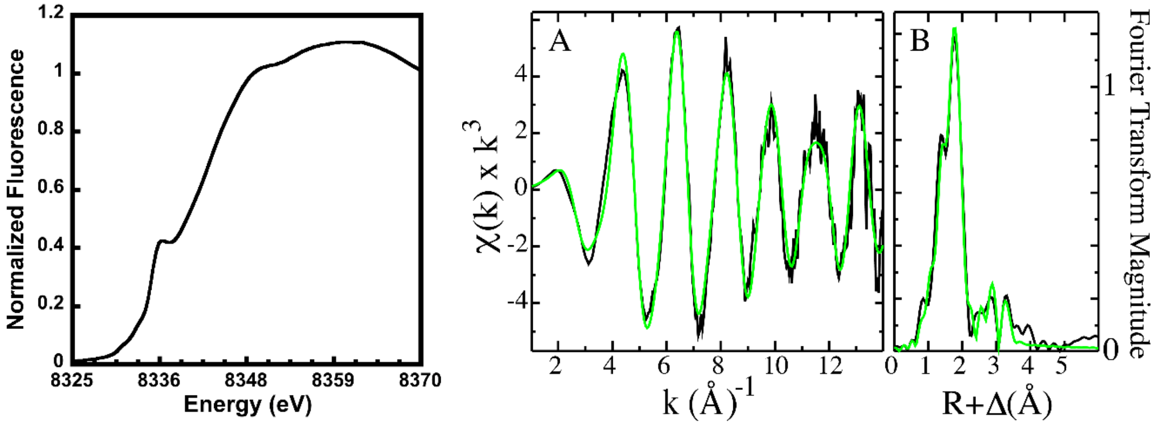

Figure S3. (*Left*) XANES spectrum of complex **1**. (*Right*) EXAFS (panel A) and Fourier transforms (panel B) of the EXAFS data for **1**. Raw EXAFS/Fourier transform data (black) and best fits (green) for **1**.

Table S4. Summary of best-fit simulations to Ni EXAFS for **1**[a].

Sample	Nearest Neighbor Ligand Environment[b]				Long Range Ligand Environment[b]								
	**Atom**[c]	**R(A)**[d]	**CN**[e]	σ^2[f]	**Atom**[c]	**R(A)**[d]	**CN**[e]	σ^2[f]	**Atom**[c]	**R(A)**[d]	**CN**[e]	σ^2[f]	***F'***[g]
Complex **1**	N	1.90	2.0	5.40	C	2.78	1.0	2.03	C	3.82	3.5	4.22	0.34
	S	2.17	2.0	2.72	C	3.33	1.5	1.50					

[a] Data were fit over a *k* range of 1 to 14.0 $Å^{-1}$.
[b] Independent metal-ligand scattering environment.
[c] Scattering atoms: N (nitrogen), C (carbon), Oxygen (O).
[d] Average metal-ligand bond length.
[e] Average metal-ligand coordination number.
[f] Average Debye-Waller factor in $Å^2 \times 10^3$.
[g] Number of degrees of freedom weighted mean square deviation between data and fit.

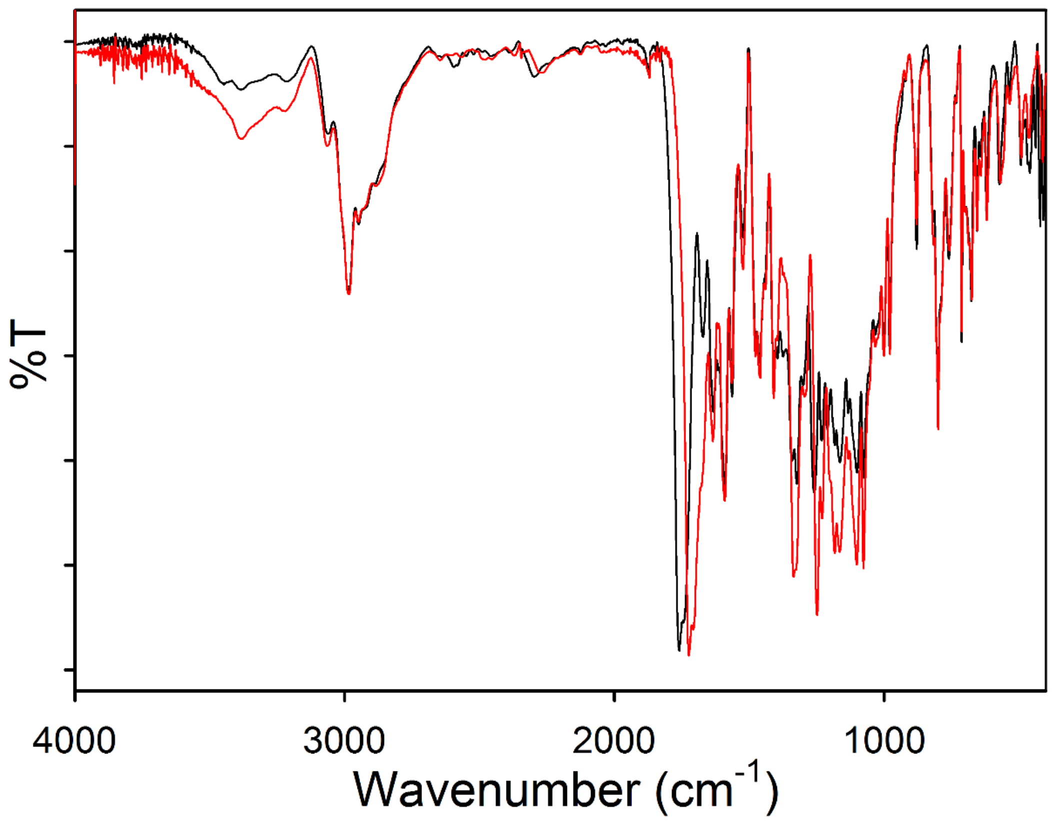

Figure S4. FTIR spectra of **2** (black) and **2-^{15}NO** (red) in a KBr matrix.

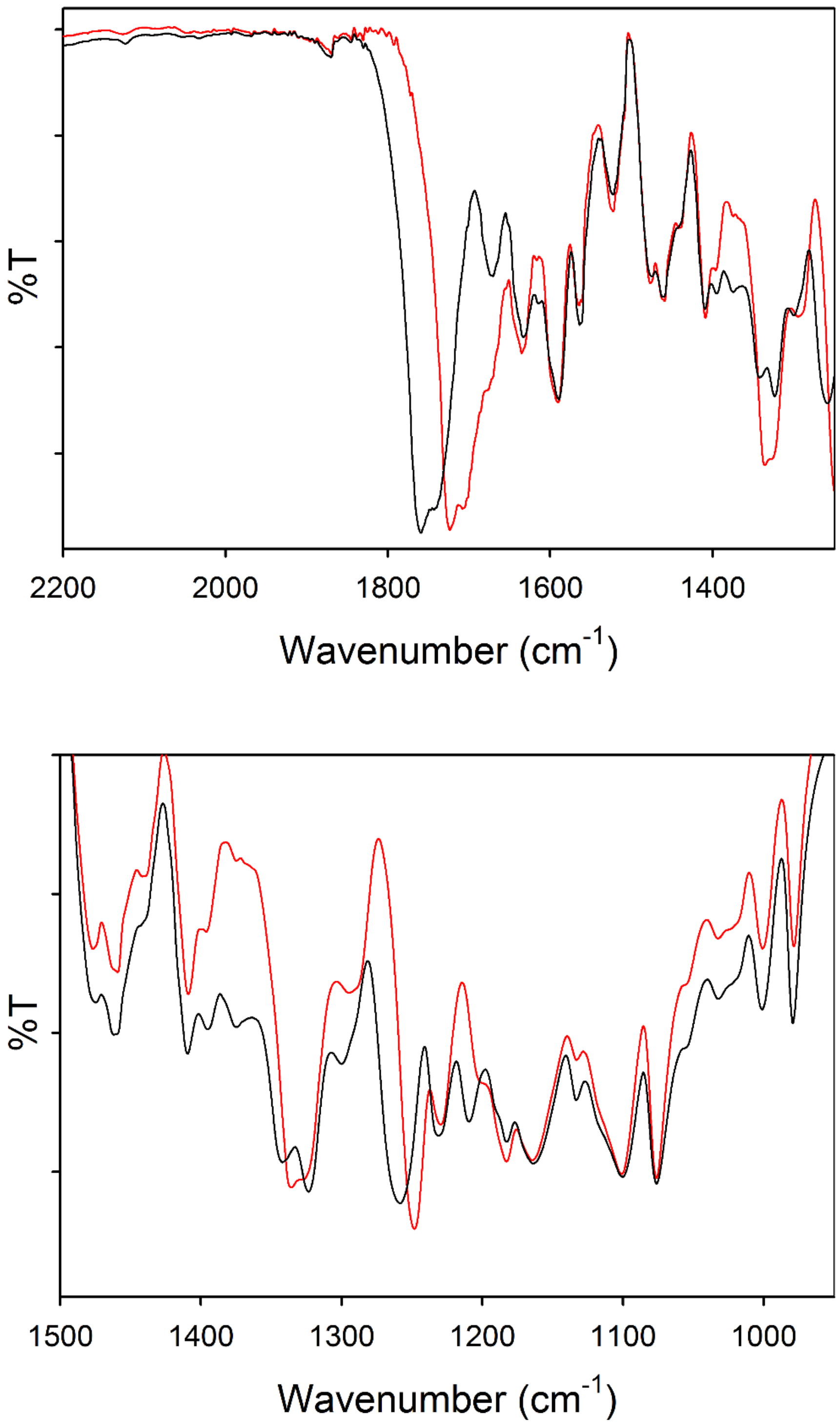

Figure S5. Zoom-in regions of the FTIR of **2** (black) and **2-^{15}NO** (red) in a KBr matrix. *Top*: Ni-NO region. *Bottom*: RNNO region.

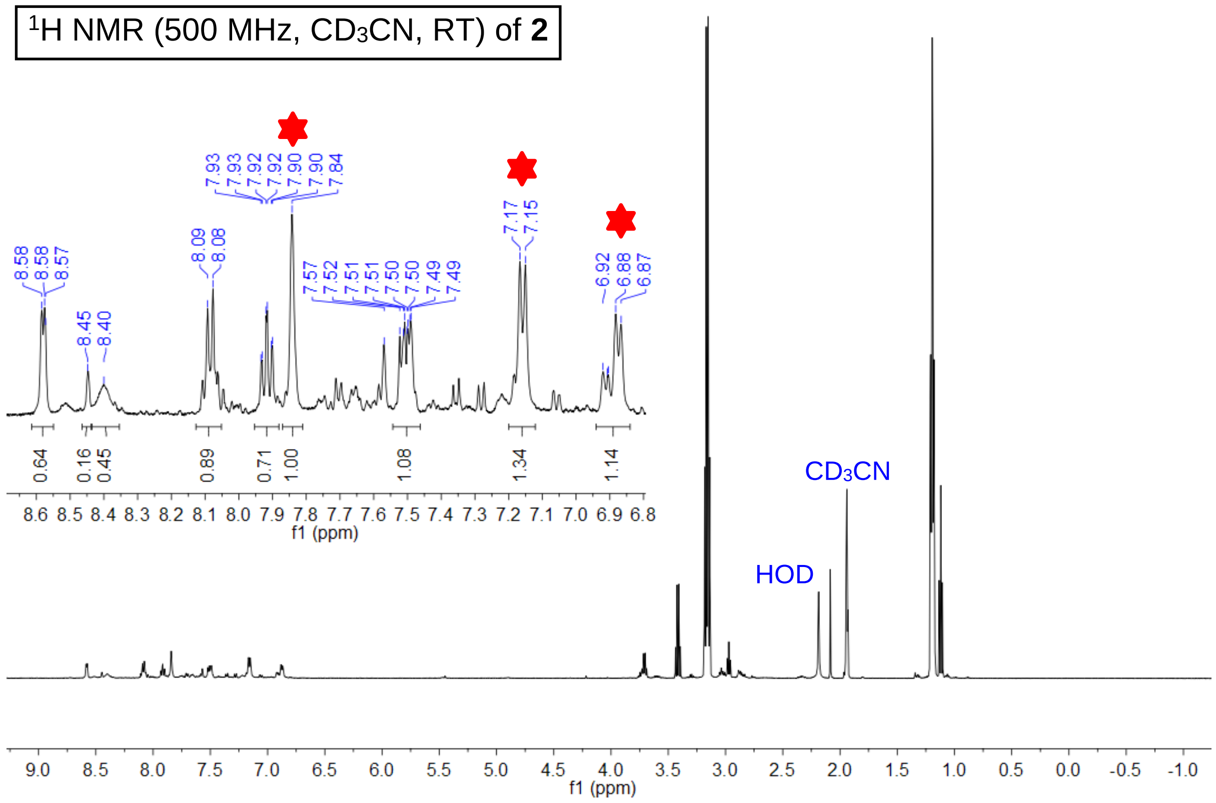

Figure S6. ^1H NMR spectrum of **2** in CD_3CN at RT (δ vs. residual protio solvent signal). The peak at 2.19 and 1.94 ppm is from water and protio solvent, respectively. Minor amounts of Et_2O (1.12, 3.42 ppm) and acetone (2.09 ppm) are present from workup. *Inset*: expansion of the aromatic-H region. Peaks labelled with a red star are assigned to the $\{NiNO\}^{10}$ complex **2**. Other peaks in the aromatic region belong to nmpS_2 (see Figures S19-20 for independent ^1H NMR characterization), minor peaks are likely due to multiple speciation in solution due to lability of the Ni-NO bond.

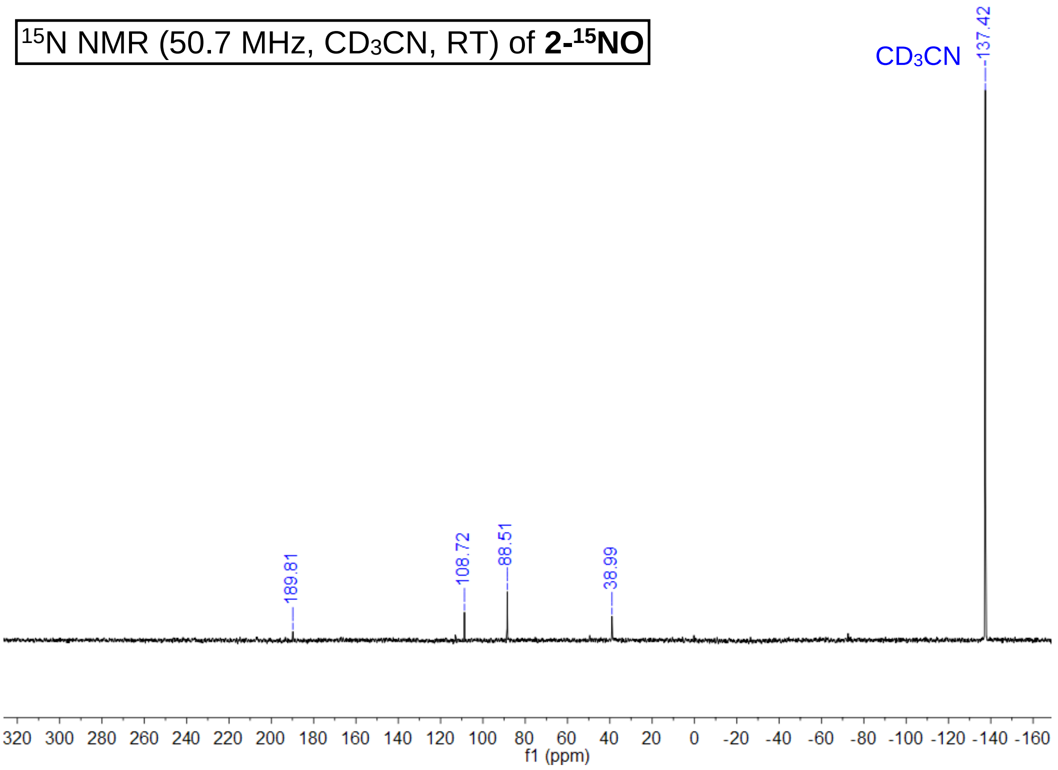

Figure S7. ^{15}N NMR spectrum of **2-^{15}NO** in CD_3CN at RT (δ vs. CH_3NO_2). Signal at -137.42 ppm is from CD_3CN.

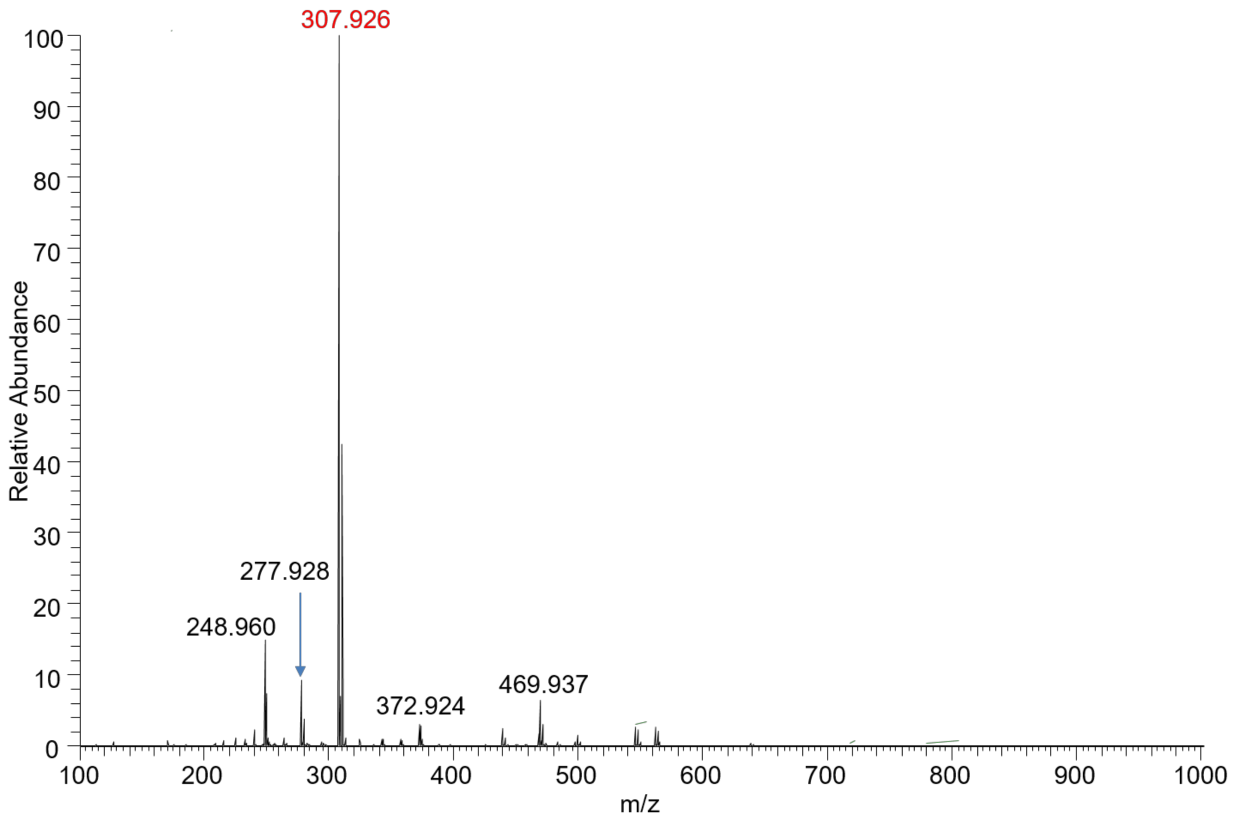

Figure S8. HR-ESI-MS(-) of **2** in MeCN. Isotopically sensitive peaks are labelled. Other than the $[M\text{-}2Et_4N]^{2-}$ peak at m/z = 307.926 (in red, zoom-in with isotopic distribution on next page, see Figure S9), the only other identifiable peak is at m/z = 248.960 attributed to the $[M\text{-}2Et_4N]^{2-}$ peak of **3**. See Figures S10-S11 for **2-^{15}NO**.

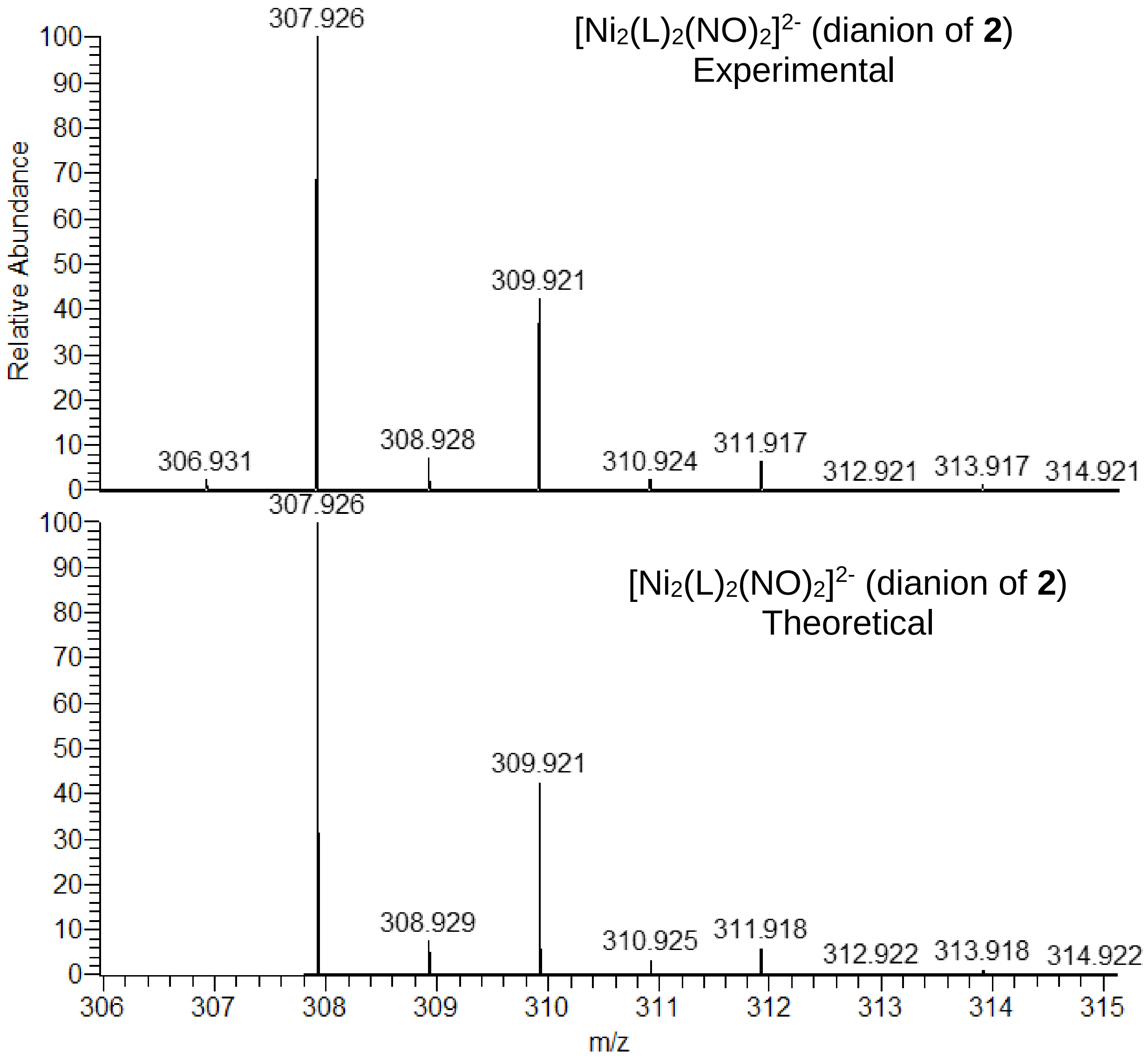

Figure S9. *Top:* Zoom-in of the HR-ESI-MS(-) of the dianion of **2** in MeCN. *Bottom*: theoretical isotopic distribution.

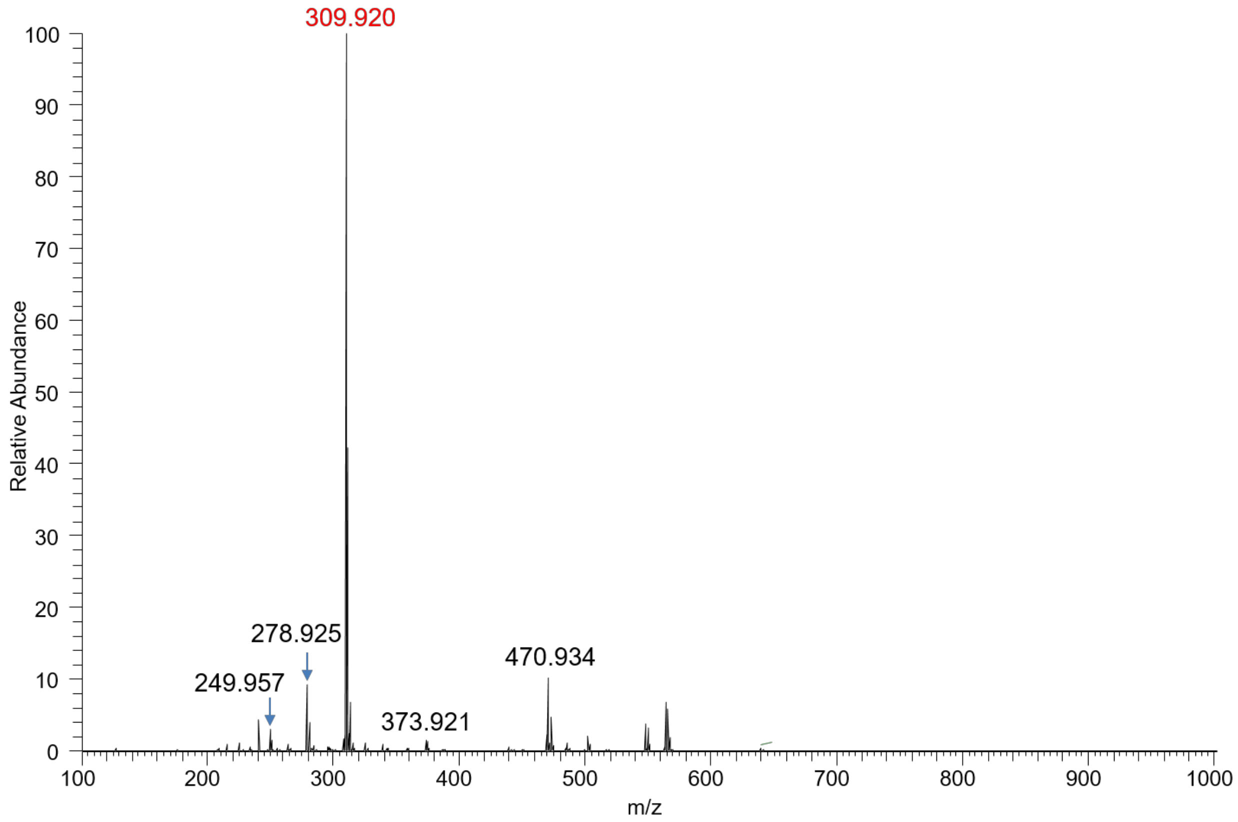

Figure S10. HR-ESI-MS(-) of **2-^{15}NO** in MeCN. Isotopically sensitive peaks are labelled. Other than the $[M\text{-}2Et_4N]^{2-}$ peak at *m*/*z* = 309.920 (in red, zoom-in with isotopic distribution on next page, see Figure S11), the only other identifiable peak is at *m*/*z* = 249.957 attributed to the $[M\text{-}2Et_4N]^{2-}$ peak of **3-^{15}NO**.

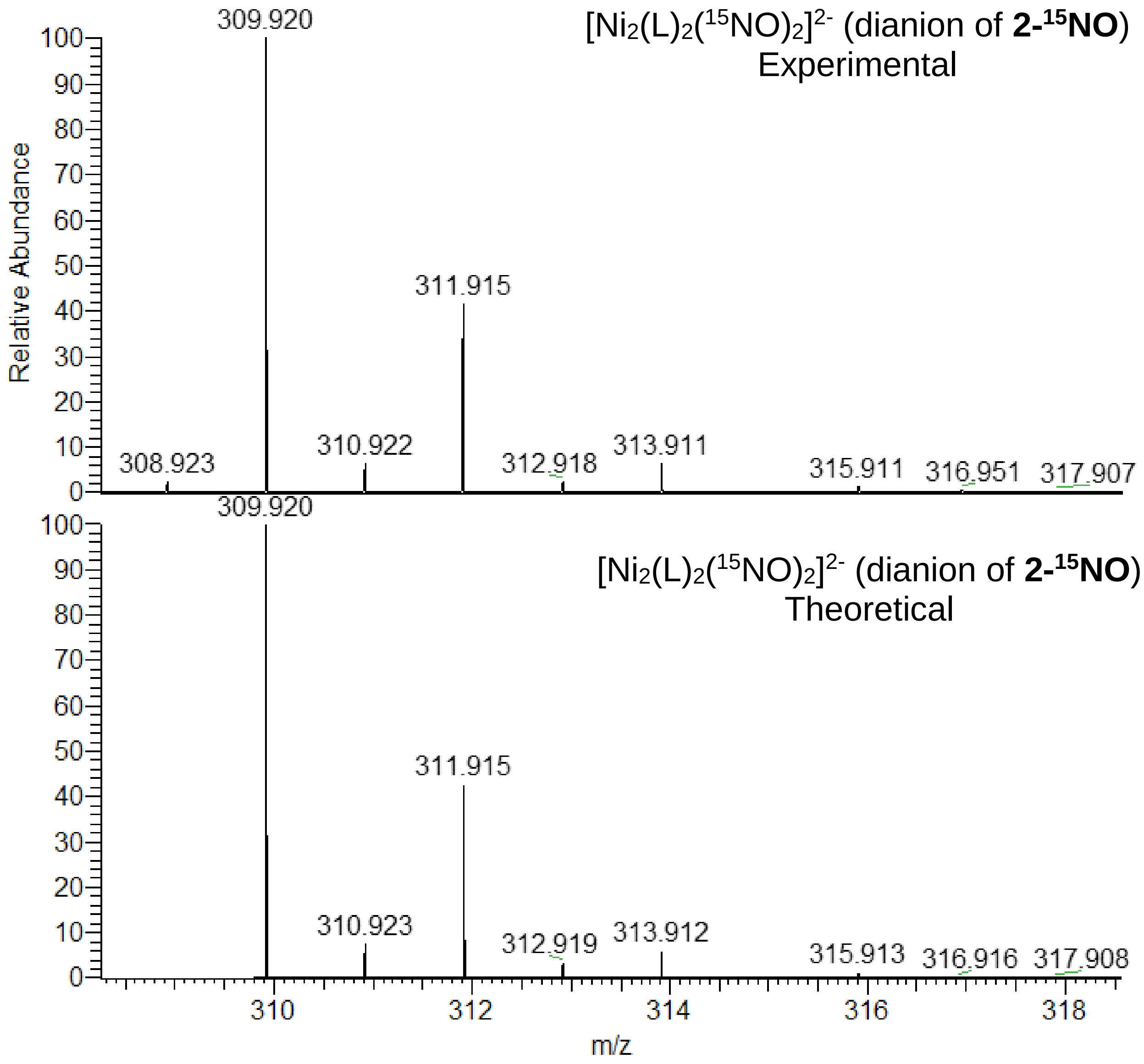

Figure S11. *Top:* Zoom-in of the HR-ESI-MS(-) of the dianion of **2-^{15}NO** in MeCN. *Bottom*: theoretical isotopic distribution.

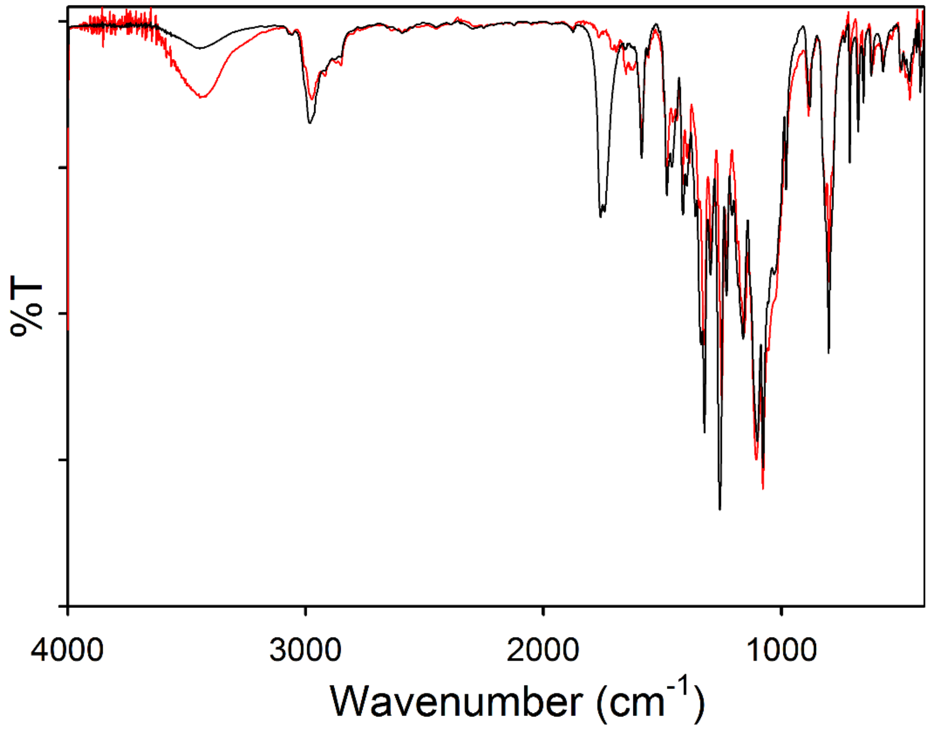

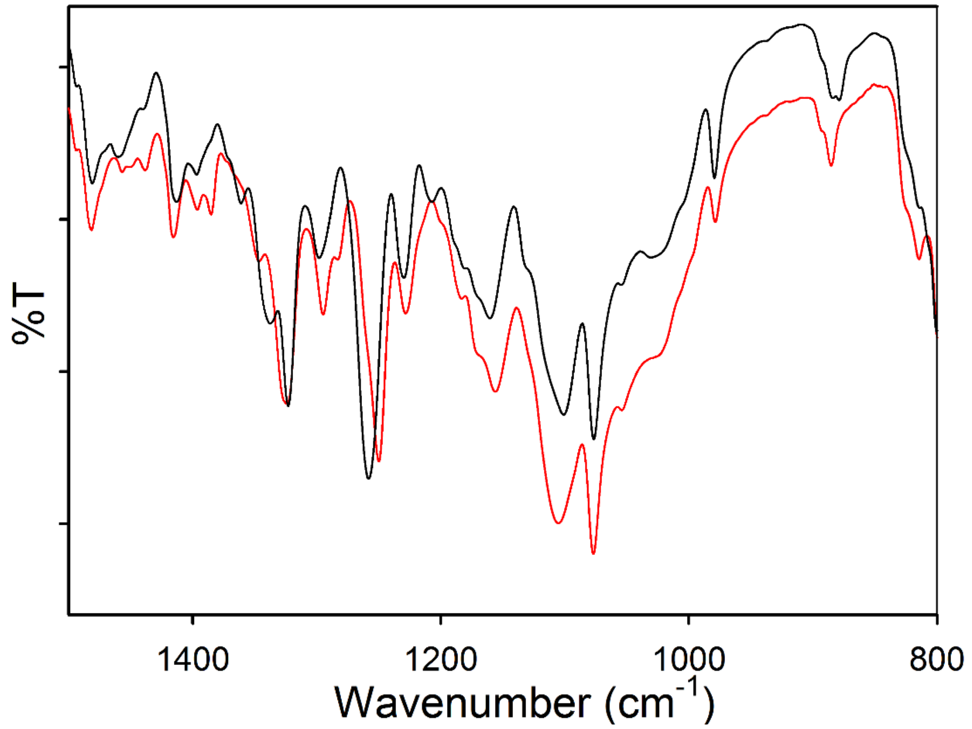

Figure S12. FTIR spectra of **3** (black) and **3-^{15}NO** (red) in a KBr matrix. *Top*: full spectrum. *Bottom*: zoom-in of isotopically-shifted areas.

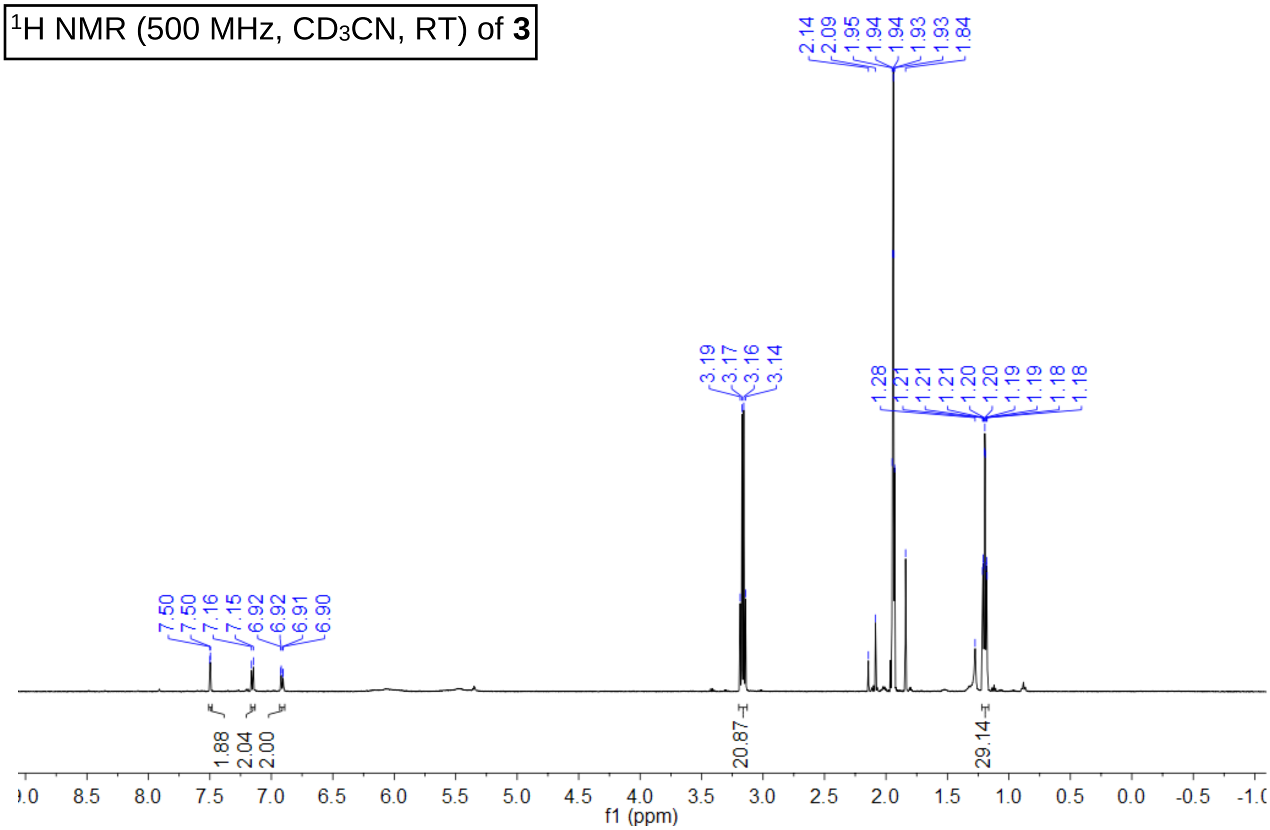

Figure S13. ¹H NMR spectrum of **3** in CD_3CN at RT (δ vs. residual protio solvent signal).

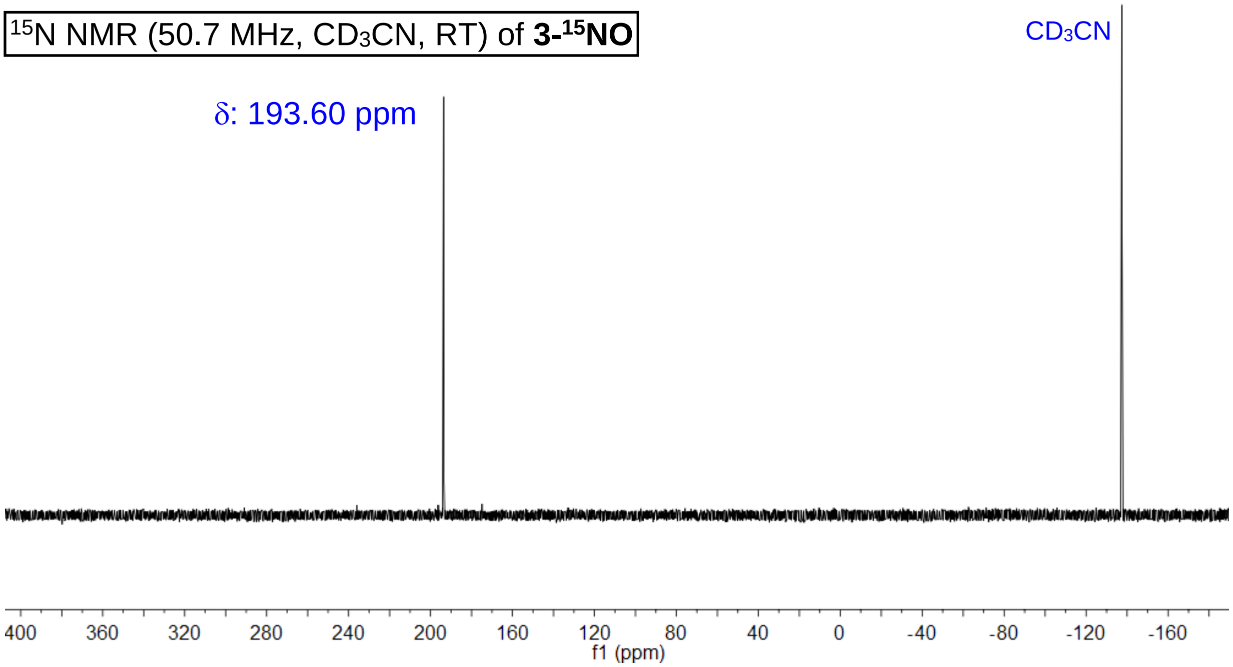

Figure S14. ^{15}N NMR spectrum of **3-^{15}NO** in CD_3CN at RT (δ vs. CH_3NO_2). Signal at -137.42 ppm is from CD_3CN.

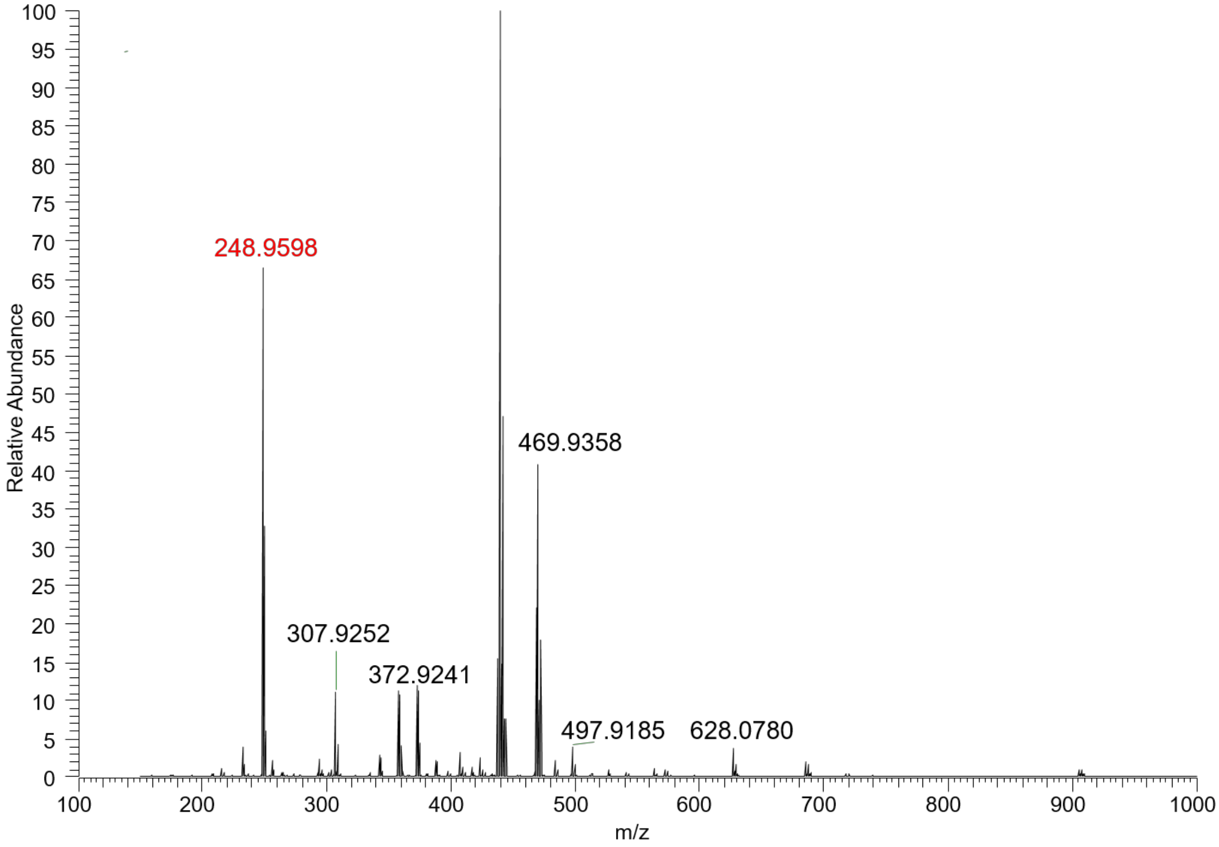

Figure S15. HR-ESI-MS(-) of **3** in MeCN. Isotopically sensitive peaks are labelled. Other than the $[M\text{-}2Et_4N]^{2-}$ peak of **3** at *m*/*z* = 248.960 (in red, zoom-in with isotopic distribution on next page, see Figure S16), the other identifiable peaks are at *m*/*z* = 307.925 attributed to the $[M\text{-}2Et_4N]^{2-}$ peak of **2**, and the $[M\text{-}Et_4N]^{-}$ peak of **3** at *m*/*z* = 628.078. See Figures S17-S18 for **3-^{15}NO**.

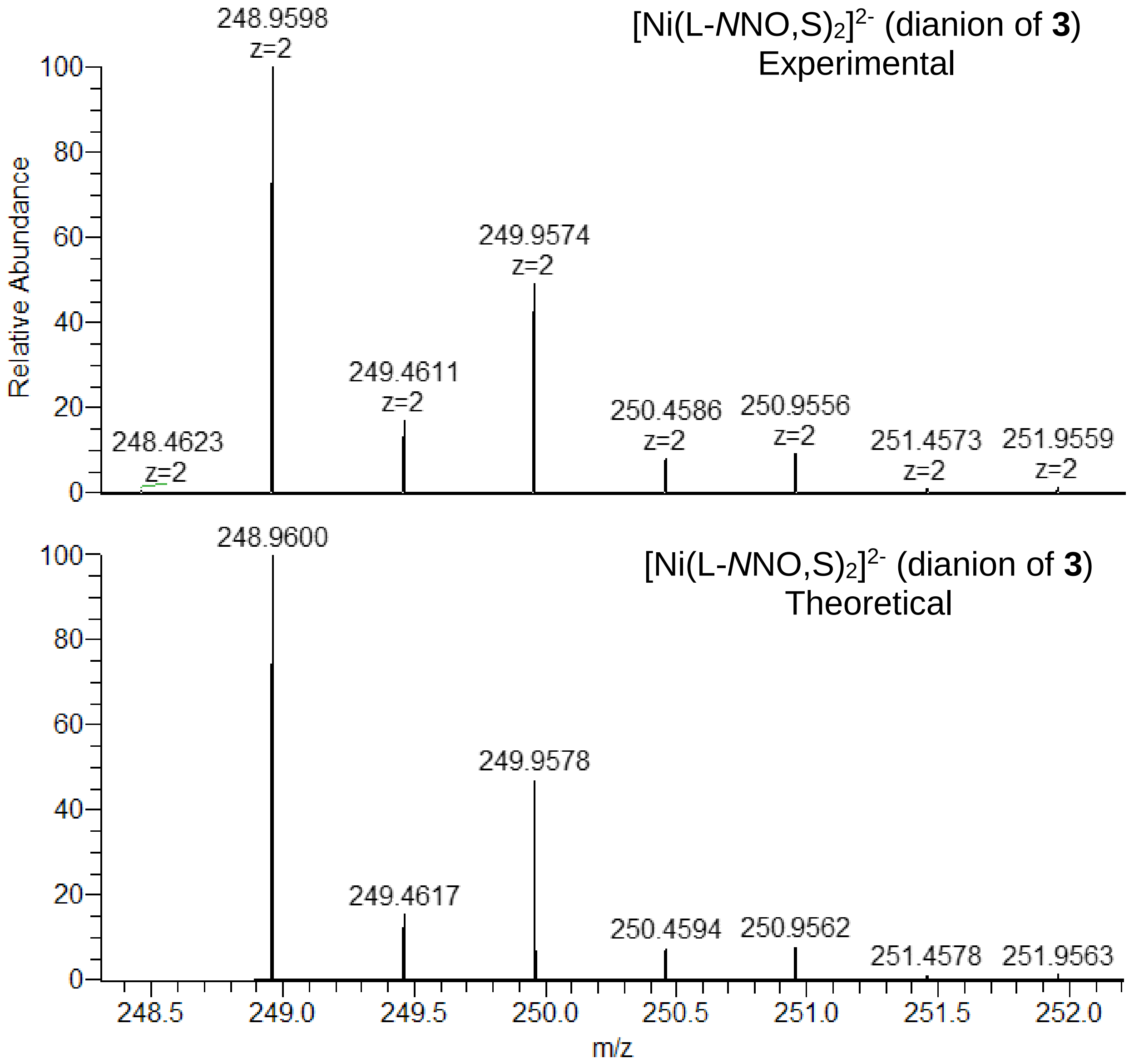

Figure S16. *Top:* Zoom-in of the HR-ESI-MS(-) of the dianion of **3** in MeCN. *Bottom*: theoretical isotopic distribution.

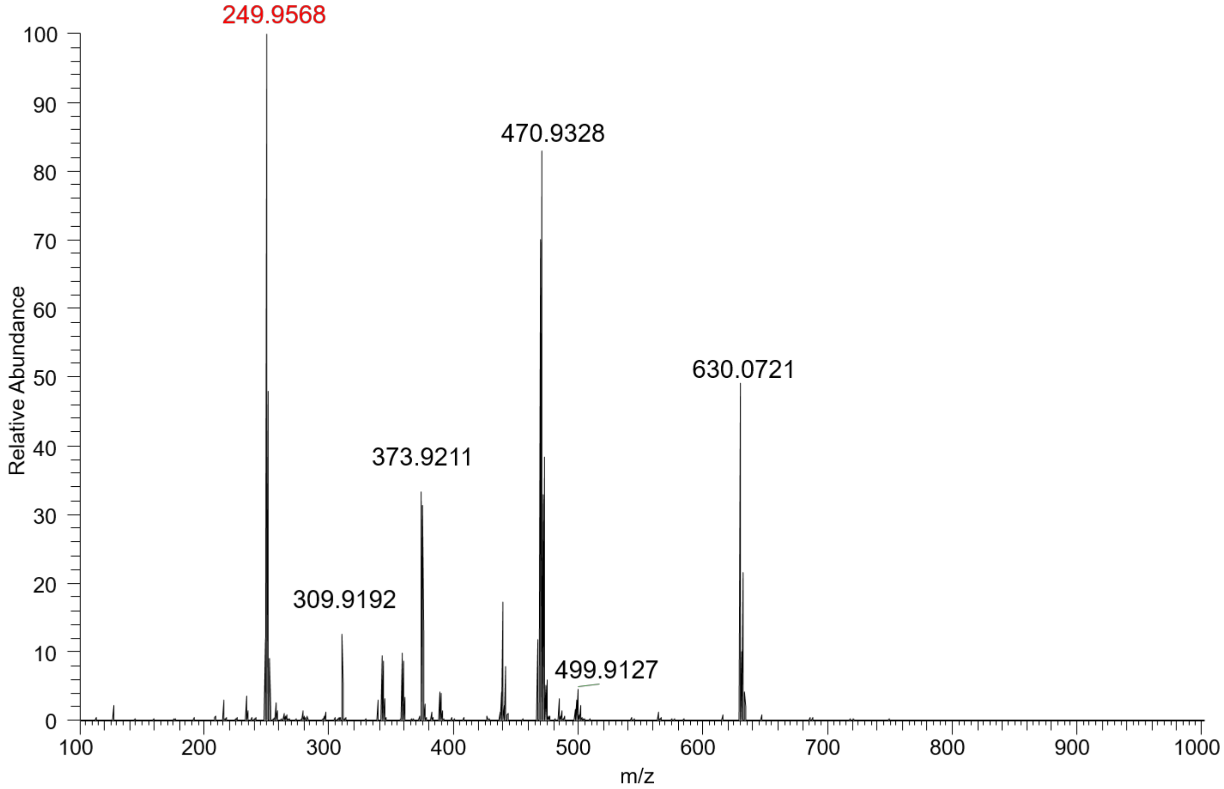

Figure S17. HR-ESI-MS(-) of **3-^{15}NO** in MeCN. Isotopically sensitive peaks are labelled. Other than the $[M-2Et_4N]^{2-}$ peak of **3-^{15}NO** at *m*/*z* = 248.957 (in red, zoom-in with isotopic distribution on next page, see Figure S18), the other identifiable peaks are at *m*/*z* = 309.919 attributed to the $[M-2Et_4N]^{2-}$ peak of **2-^{15}NO**, and the $[M-Et_4N]^{-}$ peak of **3-^{15}NO** at *m*/*z* = 630.072.

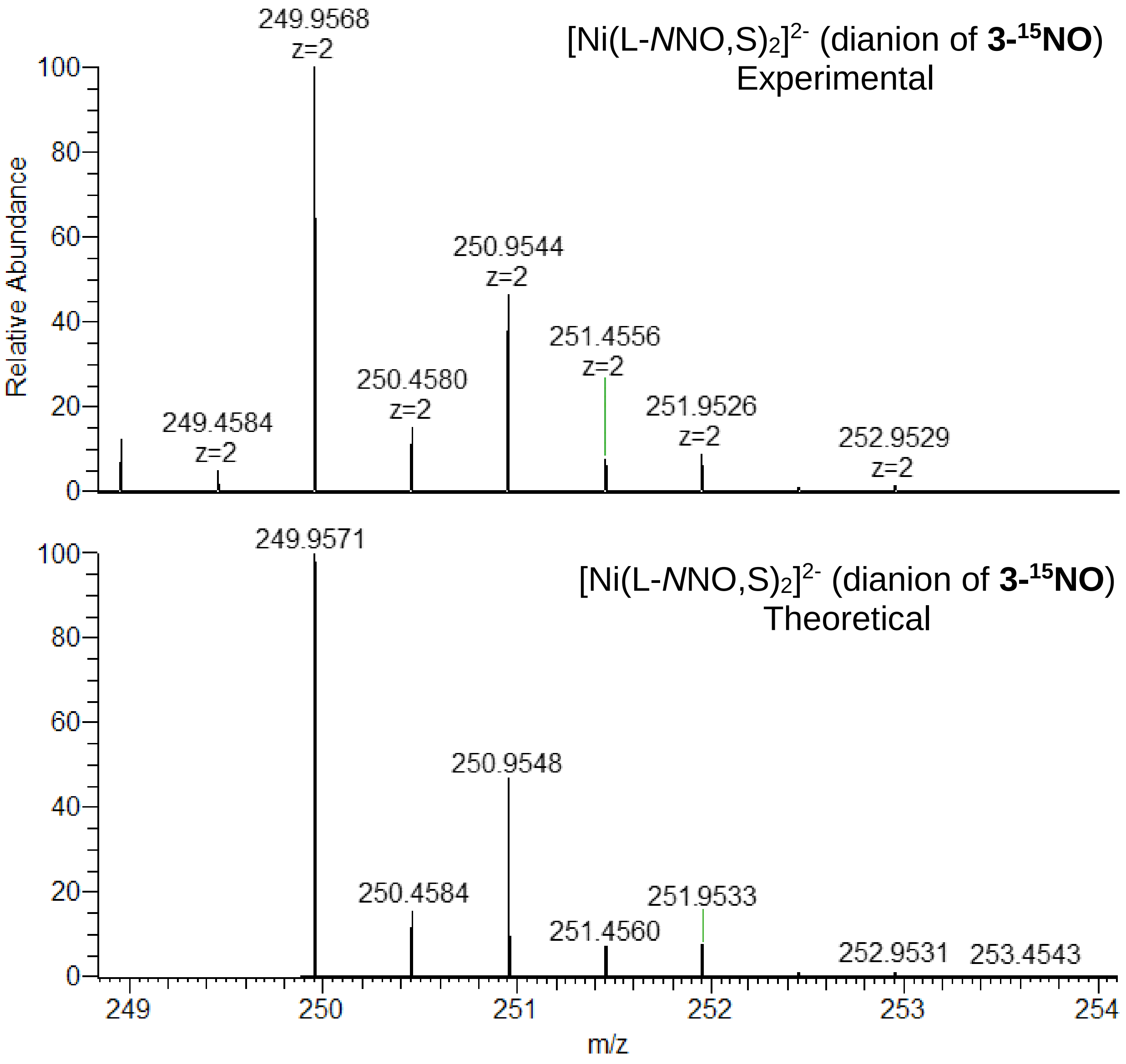

Figure S18. *Top:* Zoom-in of the HR-ESI-MS(-) of the dianion of **3-^{15}NO** in MeCN. *Bottom*: theoretical isotopic distribution.

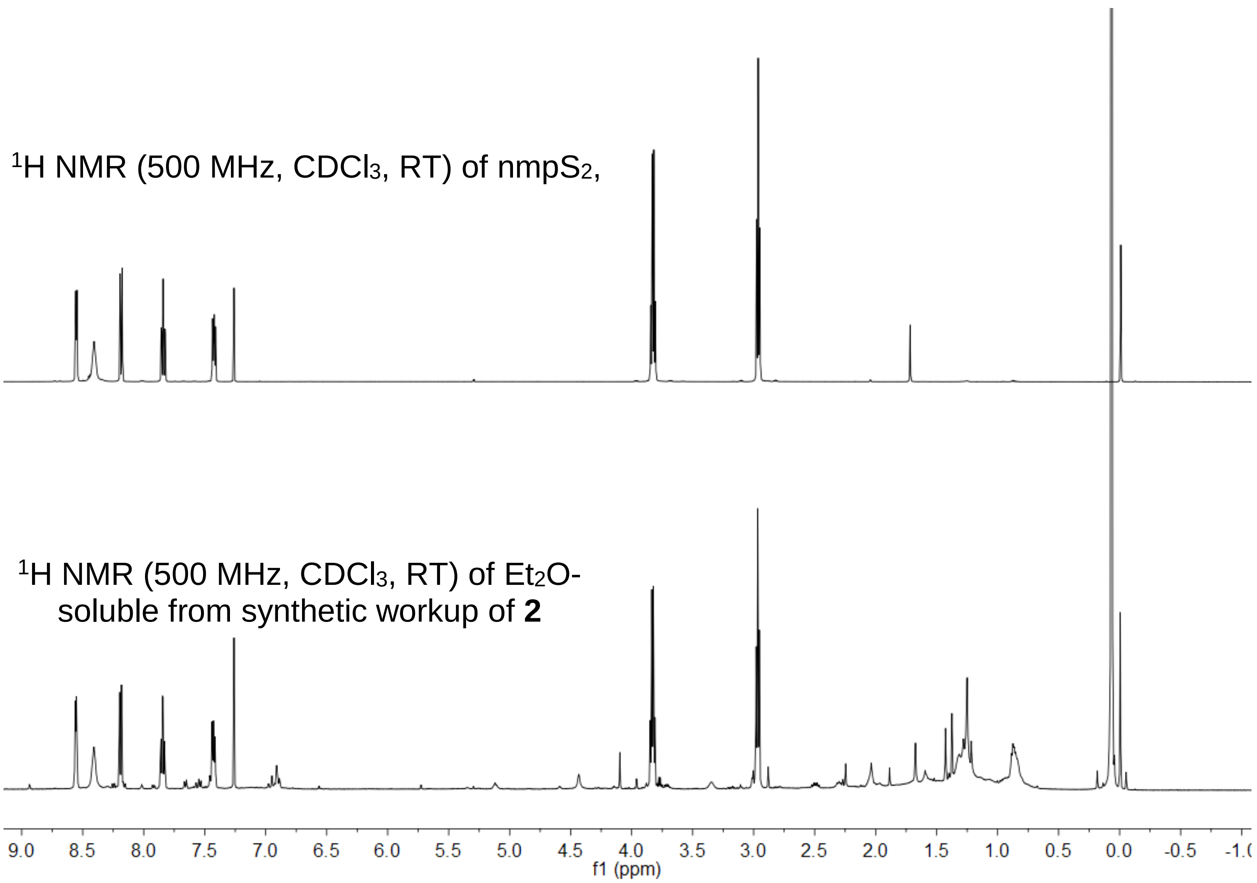

Figure S19. ^1H NMR spectrum of the Et_2O-soluble material from the synthetic workup of **2** (bottom) compared to independently synthesized nmp-disulfide ($nmpS_2$, top) in $CDCl_3$.

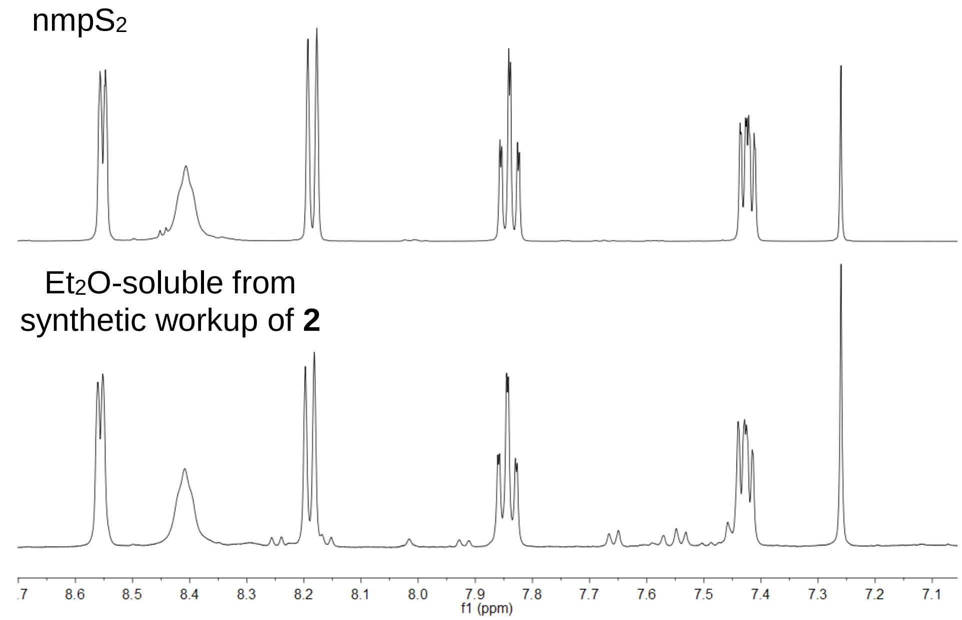

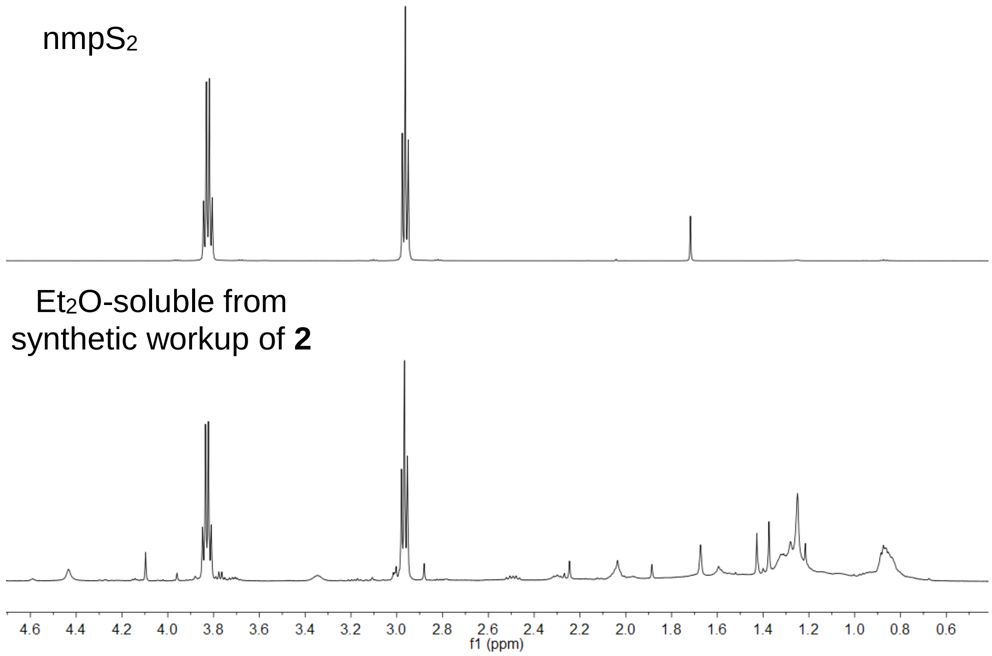

Figure S20. Zoom-in from Figure S19 of the aromatic (top) and aliphatic (bottom) of the ^{1}H NMR spectrum of the Et_2O-soluble material from workup of **2** compared to independently synthesized $nmpS_2$ in $CDCl_3$.

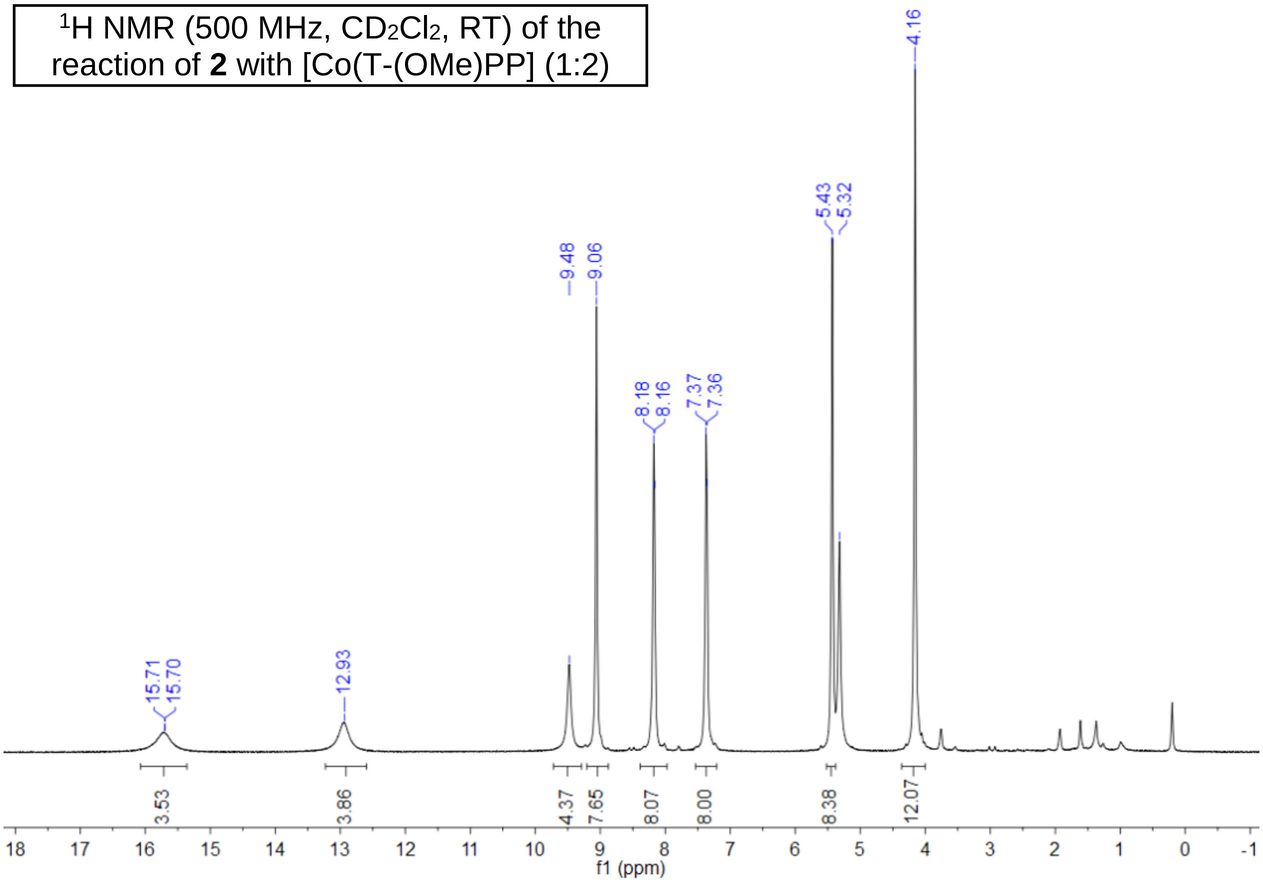

Figure S21. ^1H NMR spectrum of the MeOH-insoluble portion of the reaction of **2** with [Co(T-(OMe)PP)] (1:2) in CD_2Cl_2.

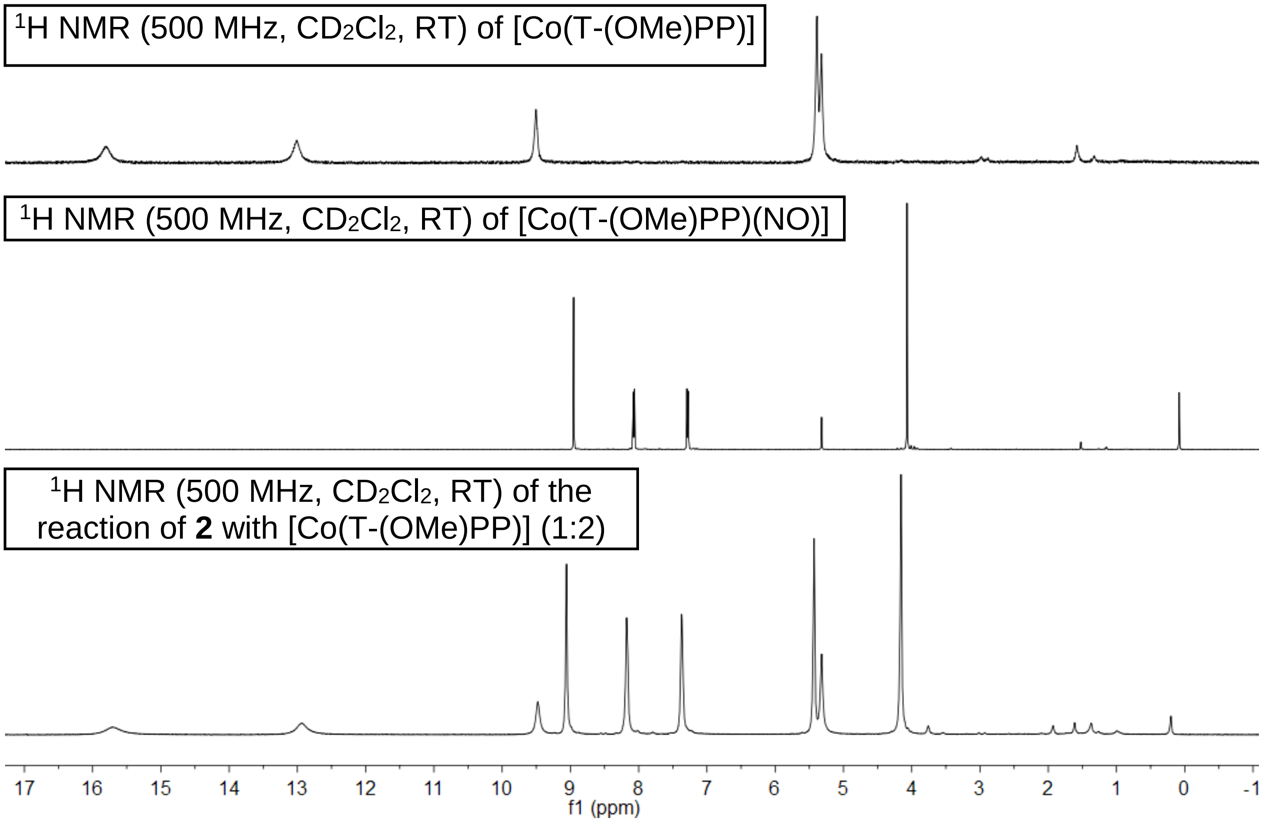

Figure S22. ^{1}H NMR spectra (all in CD_2Cl_2) of [Co(T-(OMe)PP)] (top), [Co(T-(OMe)PP)(NO)] (middle), and the MeOH-insoluble portion of the reaction of **2** with [Co(T-(OMe)PP)] (1:2) (bottom, same spectrum as in Figure S21).

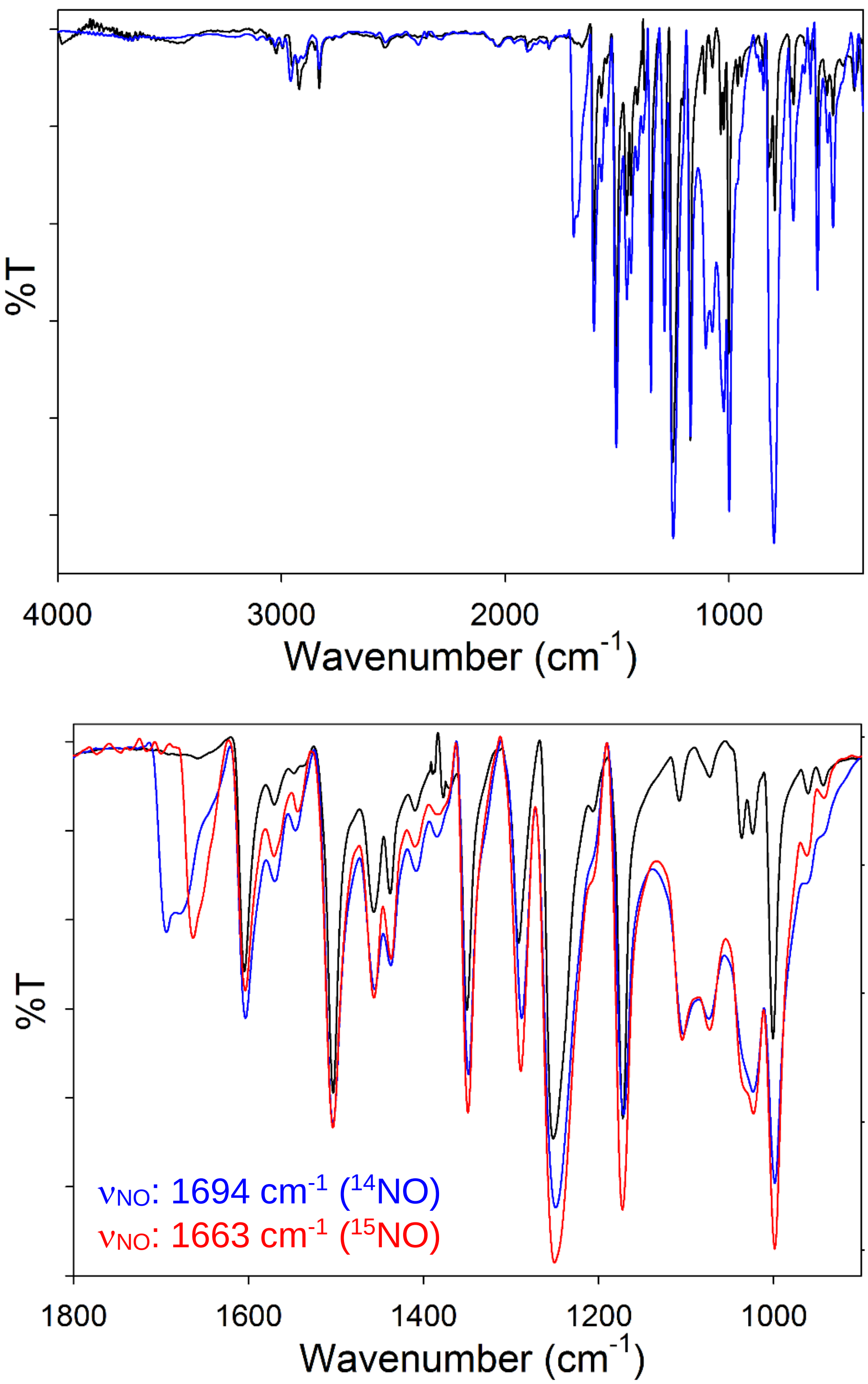

Figure S23. *Top*: Solid-state FTIR spectrum of the MeOH-insoluble portion of the reaction of **2** with [Co(T-(OMe)PP)] (1:2) (blue) compared to [Co(T-(OMe)PP)] (black). *Bottom*: zoom-in of the IR spectra above (same color coding) with the addition of the MeOH-insoluble portion of the reaction of **2-^{15}NO** with [Co(T-(OMe)PP)] (1:2) (red). All measured in a KBr matrix.

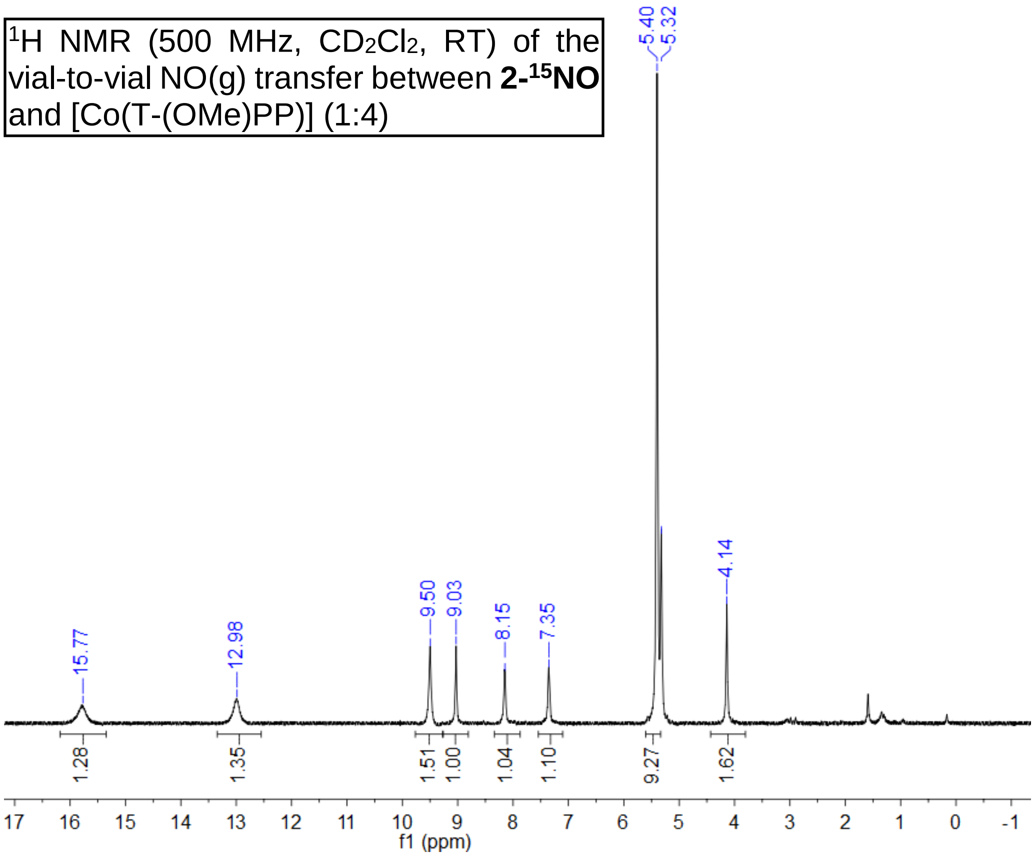

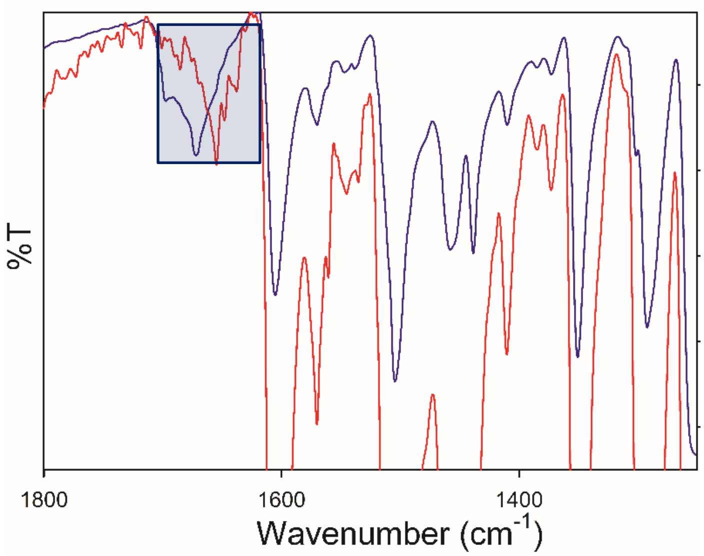

Figure S24. *Top*: ^{1}H NMR spectrum of the outer vial (5-dram) contents used in the NO(g) vial-to-vial reaction of **2-^{15}NO** with [Co(T-(OMe)PP)] (1:4) in CD_2Cl_2. See Fig. S22 (pg. S35) for the ^{1}H NMR of [Co(T-(OMe)PP)] and [Co(T-(OMe)PP)(NO)]. *Bottom*: Solid-state FTIR spectrum of the outer vial (5-dram) contents of the **2**/[Co(T-(OMe)PP)] (1:3) (blue) and **2-^{15}NO**/[Co(T-(OMe)PP)] (1:4) (red) reaction. The N-O stretch of the CoP-nitrosyl is highlighted in the blue box. All measured in a KBr matrix.

Computational details

Density functional theory (DFT) calculations were performed with the ORCA electronic structure package, version 3.0.3.[18] Geometry optimization and frequency analysis of all models were performed using the BP86 functional[19, 20] along with resolution-of-the-identity (RI) approximation[21] and Grimme's D3(BJ) dispersion correction[22, 23] with coordinates from the crystal structures of **2** and **3**. Ahlrich's triple-ζ basis set def2-TZVPP and the auxiliary basis set def2-TZVPP/J were selected for Ni, S, N, and O atoms, while Ahlrich's split-valence basis set def2-SV(P) and the auxiliary basis set def2-SVP/J were selected for the remaining atoms.[24, 25] The integration grid was set at GRID5 with NOFINALGRID.

Single-point energy (SPE) calculations were performed on the optimized structures using the OLYP functional (OPTX exchange functional of Cohen and Handy[26] coupled with Lee, Yang, and Parr's correlation functional[27]) with RI approximation.[21] Ahlrich's triple-ζ basis set def2-TZVPP and the auxiliary basis set def2-TZVPP/J were selected for Ni, S, N, and O atoms, while Ahlrich's split valence basis set def2-SV(P) and the auxiliary basis set def2-SVP/J were selected for the remaining atoms.[24, 25] The integration grid was set at GRID5 with FINALGRID7. The conductor-like screening model (COSMO)[28] was utilized to model solvent environments of DMF (ε = 38.3, n = 1.430). UCSF Chimera[29] was used to generate model structures and to visualize isosurface plots of MOs and spin-density plots with isodensity values of 0.05 a.u., respectively. Natural population analysis (NPA) was performed with JANPA to obtain the atomic charges.[30]

Table S5. Löwdin population analysis derived from the DFT calculations for selected MOs of **2*** from OLYP/def2-TZVPP with COSMO(DMF). Only s and p contributions were tabulated for N, S, and O atoms.

MO Label	MO #	Energy eV	Energy kcal/mol	Energy kJ/mol	Ni1	Ni1'	S1	S1'	N2	O1	N2'	O1'	N3	O2	N1	N3'	O2'	N1'
HOMO-4	150	-4.313	-416.13	-99.46	32.4	32.3	5.5	5.6	0.8	1.0	1.0	1.0	1.0	2.8	1.7	0.9	2.6	1.8
HOMO-3	151	-4.1198	-397.49	-95.00	10.7	10.5	1.4	1.4	0.6	0.2	0.6	0.3	8.4	20.9	2.4	8.5	21.1	2.5
HOMO-2	152	-4.0253	-388.37	-92.82	3.1	3.3	0.2	0.1	1.0	1.0	1.1	1.1	11.9	23.3	4.0	11.9	23.1	4.0
HOMO-1	153	-3.6324	-350.47	-83.76	23.1	23.2	8.2	8.2	4.1	3.8	4.2	3.7	2.6	2.7	2.4	2.7	2.7	2.4
HOMO	154	-3.4997	-337.66	-80.70	19.0	19.0	9.7	9.6	3.8	3.1	3.7	3.1	2.0	3.3	1.3	2.1	3.3	1.4
LUMO	155	-2.2281	-214.97	-51.38	13.3	17.6	4.0	3.6	12.8	7.6	17.7	10.7	0.1	0.1	0.1	0.1	0.1	0.1
LUMO+1	156	-2.219	-214.10	-51.17	15.4	11.3	2.8	3.1	19.7	11.7	14.8	8.8	0.4	0.4	0.1	0.4	0.3	0.0
LUMO+2	157	-2.0388	-196.71	-47.01	16.1	15.9	3.2	3.2	15.4	8.9	15.1	8.8	0.7	0.4	1.6	0.7	0.4	1.6
LUMO+3	158	-2.0052	-193.47	-46.24	15.6	15.9	2.0	2.1	16.1	9.2	16.4	9.5	0.5	0.3	1.2	0.6	0.3	1.3
LUMO+4	159	-1.5786	-152.31	-36.40	1.8	1.8	1.1	1.1	0.0	0.0	0.0	0.0	14.8	9.4	2.6	14.9	9.5	2.7

Table S6. Löwdin population analysis derived from the DFT calculations for selected MOs of **3*** from OLYP/def2-TZVPP with COSMO(DMF). Only s and p contributions were tabulated for N, S, and O atoms.

**MO Label**	**MO #**	**Energy (eV)**	**Energy (kJ/mol)**	**Energy (kcal/mol)**	**Ni**	**S1**	**S1′**	**N1**	**N1′**	**N3**	**N3′**	**O2**	**O2′**
HOMO-4	121	-4.1931	-404.56	-96.69	22.2	14.6	14.6	1.8	1.8	1.5	1.4	5.8	5.9
HOMO-3	122	-3.9978	-385.72	-92.19	2.3	1.0	1.0	4.4	4.4	13.1	13.1	23.7	23.8
HOMO-2	123	-3.9032	-376.59	-90.01	77.8	0.3	0.4	1.8	1.8	1.8	1.9	3.9	3.8
HOMO-1	124	-3.7348	-360.34	-86.12	93.2	1.8	1.8	0.8	0.7	0.0	0.0	0.0	0.1
HOMO	125	-3.3704	-325.19	-77.72	51.3	13.5	13.4	0.5	0.5	0.9	0.9	1.4	1.4
LUMO	126	-1.8682	-180.25	-43.08	45.6	10.0	10.0	5.1	5.1	2.7	2.7	2.2	2.2
LUMO+1	127	-1.6311	-157.37	-37.61	6.7	1.0	1.0	3.9	3.9	15.9	16.0	9.7	9.7
LUMO+2	128	-1.4501	-139.91	-33.44	4.3	0.2	0.2	4.0	3.9	13.7	13.7	8.3	8.3
LUMO+3	129	-0.9557	-92.21	-22.04	1.0	3.2	3.1	0.3	0.3	0.1	0.1	0.1	0.1
LUMO+4	130	-0.9493	-91.59	-21.89	1.7	3.9	4.0	0.0	0.0	0.1	0.1	0.1	0.1

Table S7. Optimized BP86/def2-TZVPP Cartesian coordinates (Å) for **2***.

Ni	2.32294	1.46887	2.01801
Ni	0.74252	-1.05358	3.15687
S	2.51096	-0.78594	1.64005
S	0.16446	1.14728	2.87442
N	3.29329	2.36849	2.99972
O	3.91698	2.93142	3.84383
N	1.19799	-1.75295	4.57750
O	1.67947	-2.15490	5.59001
N	1.77530	2.75687	-0.58949
O	2.03457	3.80933	0.04081
N	1.85380	1.63341	0.10757
O	-0.67612	-3.62714	2.24322
N	-1.17557	-2.67762	1.59416
N	-0.68499	-1.47653	1.86082
C	-0.02444	-0.71613	-3.88019
C	0.53235	-0.74045	-2.49126
C	-3.42707	0.24600	-1.94166
C	0.74069	-1.97091	-1.83117
C	0.90007	0.46968	-1.87870
C	-2.48456	0.47449	-0.80184
C	1.34612	-1.97769	-0.57004
C	1.50482	0.47088	-0.60306
C	-2.09748	1.78625	-0.45181
C	-2.04007	-0.62597	-0.04932
C	1.74951	-0.77630	0.04896
C	-1.28881	1.98470	0.67237
C	-1.21878	-0.43371	1.08261
C	-0.85640	0.89608	1.45792
H	0.40962	-2.91241	-2.29579
H	0.70097	1.43287	-2.36615
H	-2.41150	2.64091	-1.07057
H	-2.30057	-1.65174	-0.34072
H	1.48729	-2.92274	-0.02236
H	-0.94704	2.99584	0.94405
F	0.96578	-0.66933	-4.83978
F	-0.81080	0.36713	-4.12522
F	-0.76296	-1.82198	-4.18214
F	-3.41592	1.25547	-2.85851
F	-3.18261	-0.90922	-2.61801
F	-4.73866	0.15084	-1.52441

Table S8. Optimized BP86/def2-TZVPP Cartesian coordinates (Å) for **3***.

Ni	5.15626419645075	5.73000143447540	2.65256606983923
S	5.71898199341074	7.06829050921663	4.33399881023915
S	4.81982407986698	4.07101371252986	1.21361268214357
F	11.86366508930930	8.79402873525037	2.26880548257088
F	11.97705528995026	6.65169468127439	1.88519491355790
F	12.44470250549220	7.44345636920459	3.87596553650573
F	-0.61677190055006	1.34541085145883	4.04441372522491
F	0.59194453911371	1.60867544096425	5.83852722071719
F	0.82561248265909	-0.18825216995435	4.60346222565702
O	6.35121325211604	5.90007968938319	-0.30007960610048
O	2.89732708159747	7.06853281276642	4.46388137307899
N	7.17648308769296	6.31094396662302	0.53964034028476
N	6.82146813958084	6.28605218024334	1.81915208820659
N	2.62114170944056	5.87860535245541	4.21327194128451
N	3.48196067199455	5.18592974413385	3.47641609117272
C	11.57267420096197	7.56103424740839	2.82044971882111
C	8.37123274523044	7.74780278849911	4.85016343730625
C	9.72059961330683	7.84850283377716	4.49701190815341
C	10.14912140399545	7.40522756422056	3.22284324410590
C	9.20538052661944	6.87559347083563	2.31109589619831
C	7.84943778069210	6.78328532022601	2.66445727321597
C	7.41009324262748	7.20740859435722	3.95612557762424
C	0.65960154654627	1.17448206938525	4.54527512065324
C	3.24450787166383	1.86407644090412	1.85309707429016
C	2.29203512690291	1.19970462669426	2.63224167651289
C	1.71353979368827	1.85468157970809	3.74573303532780
C	2.09440076705995	3.18233813143718	4.05367525949491
C	3.04476092598157	3.85075580857511	3.26500076756734
C	3.65091346459206	3.19091324436151	2.15208399373889
H	8.03009707594396	8.08091328018032	5.84564315783580
H	10.45456425876184	8.25409066390083	5.21349981824437
H	9.52184632236226	6.52265221290068	1.32034414073029
H	3.70867888961399	1.35724946938810	0.98939330541199
H	2.00385273072233	0.16207033053992	2.39395956199318
H	1.65891088420163	3.70313692977530	4.91706901869126

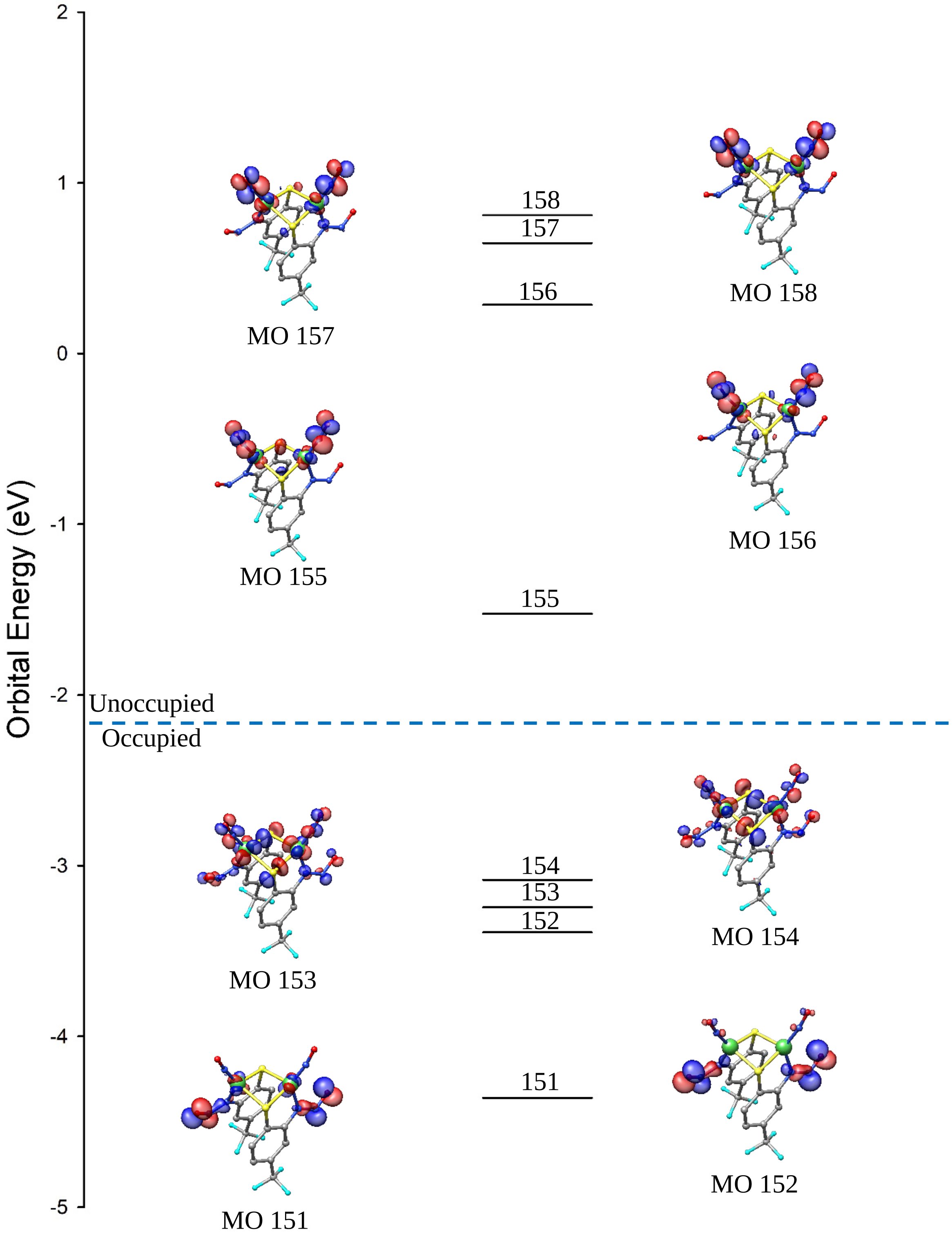

Figure S25. MO diagram of **2***. The MOs descend in the order LUMO+3, LUMO+2, LUMO+1, LUMO, HOMO, HOMO-1, HOMO-2, HOMO-3. Dashed line indicates the level below which MOs are occupied.

Table S9. Selected Bond Distances (Å) and Bond Angles (deg) from the X-ray crystal structure of **3**, compared with the DFT-optimized model **3***.

	X-ray structure **3** (avg. of two crystallographically distinct molecules)	DFT optimized structure **3***
Ni–S	2.2072	2.2217
Ni–N	1.896	1.9436
N–N	1.309	1.3281
N–O	1.264	1.247
S1–Ni–S2	165.0	168.08
N3–Ni–N1	178.0	179.47
S1–Ni–N3	94.31	93.79
S2–Ni–N3	86.0	86.28
S2–Ni–N1	93.64	93.76
S1–Ni–N1	85.52	86.28
N–N–O	115.2	117.75
τ_4	0.12	0.09

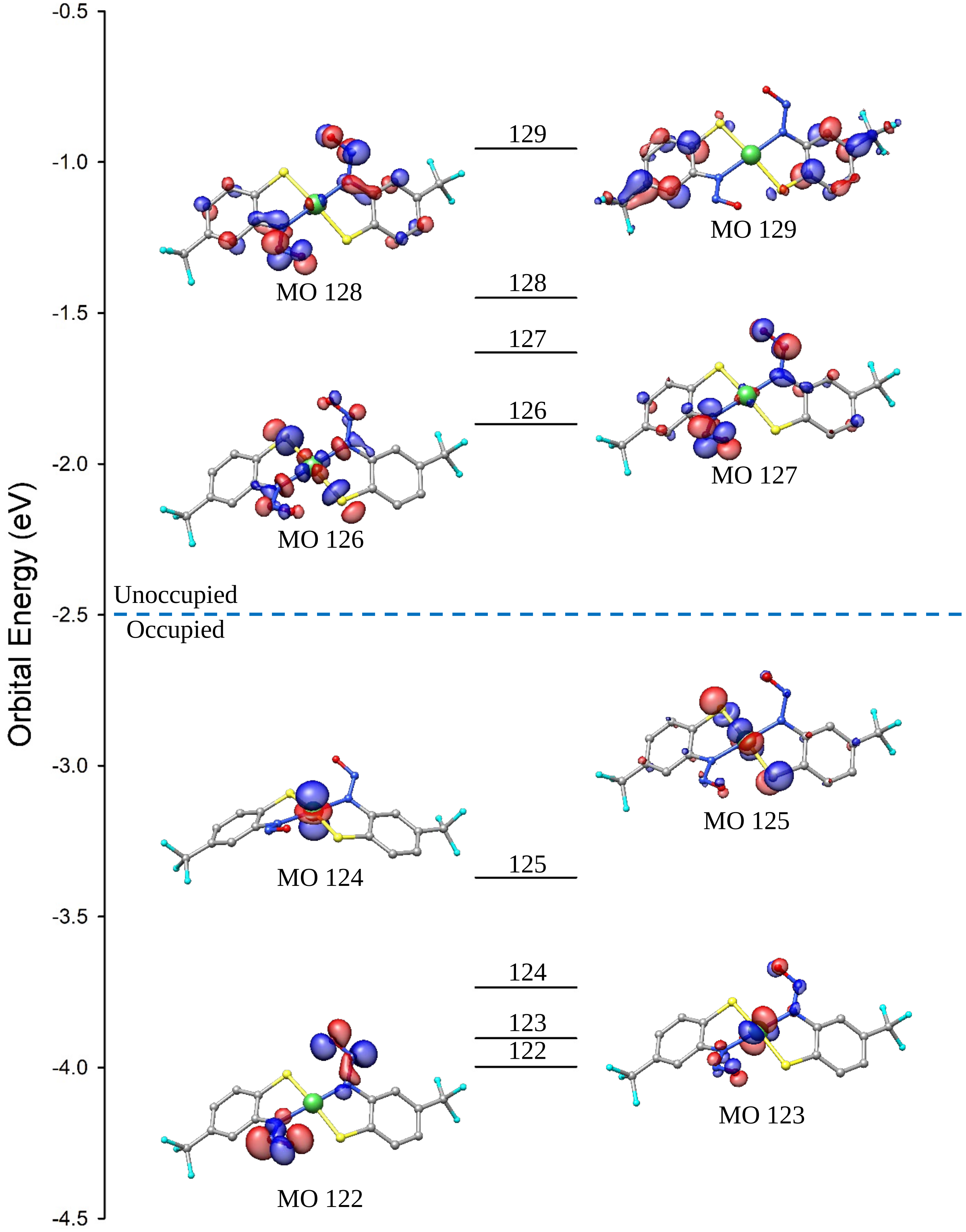

Figure S26. MO diagram of **3***. The MOs descend in the order LUMO+3, LUMO+2, LUMO+1, LUMO, HOMO, HOMO-1, HOMO-2, HOMO-3. Dashed line indicates the level below which MOs are occupied.

References

1. E. P. Broering, S. Dillon, E. M. Gale, R. A. Steiner, J. Telser, T. C. Brunold and T. C. Harrop, *Inorg. Chem.*, 2015, **54**, 3815–3828.
2. G. R. Fulmer, A. J. M. Miller, N. H. Sherden, H. E. Gottlieb, A. Nudelman, B. M. Stoltz, J. E. Bercaw and K. I. Goldberg, *Organometallics*, 2010, **29**, 2176–2179.
3. N. Walker and D. Stuart, *Acta Crystallogr.*, 1983, **A39**, 158-166.
4. G. M. Sheldrick, *Acta Crystallogr.*, 2008, **A64**, 112-122.
5. G. M. Sheldrick, *SHELXTL-2013, Crystallographic Computing System; Siemens Analytical X-Ray Instruments: Madison, WI, 2013*.
6. D. T. Cromer and J. T. Waber, in *International Tables for X-ray Crystallography*, The Kynoch Press, Birmingham, England, 1974.
7. M. N. Burnett and C. K. Johnson, *ORTEP-III, Report ORNL-6895*, Oak Ridge National Laboratory, Oak Ridge, TN, 1996.
8. G. N. George, S. J. George and I. J. Pickering, EXAFSPAK; Stanford Synchotron Radiation Lightsource: Menlo Park, CA, 2001; http://www-ssrl.slac.stanford.edu/~george/exafspak/exafs.htm.,
9. J. J. Rehr and A. L. Ankudinov, *J. Synchrotron Radiat.*, 2001, **8**, 61-65.
10. P. J. Riggs-Gelasco, T. L. Stemmler and J. E. Penner-Hahn, *Coord. Chem. Rev.*, 1995, **144**, 245-286.
11. J. J. H. Cotelesage, M. J. Pushie, P. Grochulski, I. J. Pickering and G. N. George, *J. Inorg. Biochem.*, 2012, **115**, 127-137.
12. K. Z. Bencze, K. C. Kondapalli and T. L. Stemmler, in *Applications of Physical Methods in Inorganic and Bioinorganic Chemistry: Handbook, Encyclopedia of Inorganic Chemistry*, Wiley, Chichester, U.K., 2nd edn., 2007.
13. W. W. Gu, J. Seravalli, S. W. Ragsdale and S. P. Cramer, *Biochemistry*, 2004, **43**, 9029-9035.
14. R. A. Steiner, S. P. Dzul, T. L. Stemmler and T. C. Harrop, *Inorg. Chem.*, 2017, **56**, 2849-2862.
15. L. X. Chen, X. Y. Zhang, E. C. Wasinger, J. V. Lockard, A. B. Stickrath, M. W. Mara, K. Attenkofer, G. Jennings, G. Smolentsev and A. Soldatov, *Chem Sci*, 2010, **1**, 642-650.
16. G. J. Colpas, M. J. Maroney, C. Bagyinka, M. Kumar, W. S. Willis, S. L. Suib, N. Baidya and P. K. Mascharak, *Inorg. Chem.*, 1991, **30**, 920-928.
17. C. R. Groom, I. J. Bruno, M. P. Lightfoot and S. C. Ward, *Acta Crystallographica Section B*, 2016, **72**, 171-179.
18. F. Neese, *WIREs Comput. Mol. Sci.*, 2012, **2**, 73-78.
19. A. D. Becke, *J. Chem. Phys.*, 1986, **84**, 4524-4529.
20. J. P. Perdew, *Phys. Rev. B*, 1986, **33**, 8822-8824.
21. F. Neese, *J. Comput. Chem.*, 2003, **24**, 1740-1747.
22. S. Grimme, J. Antony, S. Ehrlich and H. Krieg, *J. Chem. Phys.*, 2010, **132**, 154104.
23. S. Grimme, S. Ehrlich and L. Goerigk, *J. Comput. Chem.*, 2011, **32**, 1456-1465.
24. F. Weigend and R. Ahlrichs, *Phys. Chem. Chem. Phys.*, 2005, **7**, 3297-3305.
25. A. Schäfer, H. Horn and R. Ahlrichs, *J. Chem. Phys.*, 1992, **97**, 2571-2577.
26. A. J. Cohen and N. C. Handy, *Mol. Phys.*, 2001, **99**, 607-615.
27. C. Lee, W. Yang and R. G. Parr, *Phys. Rev. B*, 1988, **37**, 785-789.

28. A. Klamt and G. Schüürmann, *J. Chem. Soc., Perkin Trans. 2*, 1993, DOI: DOI 10.1039/p29930000799, 799-805.
29. E. F. Pettersen, T. D. Goddard, C. C. Huang, G. S. Couch, D. M. Greenblatt, E. C. Meng and T. E. Ferrin, *J. Comput. Chem.*, 2004, **25**, 1605-1612.
30. T. Y. Nikolaienko, L. A. Bulavin and D. M. Hovorun, *Comput. Theor. Chem.*, 2014, **1050**, 15-22.
